# Crosslinking of Branched PIM-1 and PIM-Py Membranes for Recovery of Toluene from Dimethyl Sulfoxide by Pervaporation

**DOI:** 10.1021/acsapm.2c01600

**Published:** 2023-01-17

**Authors:** Sulaiman Aloraini, Michael Mathias, Jessica Crone, Kurtis Bryce, Ming Yu, Richard A. Kirk, Mohd Zamidi Ahmad, Edidiong D. Asuquo, Sandra Rico-Martínez, Alexey V. Volkov, Andrew B. Foster, Peter M. Budd

**Affiliations:** † Department of Chemistry, University of Manchester, Oxford Road, ManchesterM13 9PL, United Kingdom; ‡ Department of Chemistry, College of Science and Arts, Qassim University, Ar Rass52571, Saudi Arabia; § Department of Chemical Engineering, The University of Melbourne, Melbourne, VIC3010, Australia; ∥ IU CINQUIMA, Universidad de Valladolid, Paseo Belén 5, E-47011Valladolid, Spain; ⊥ A. V. Topchiev Institute of Petrochemical Synthesis, 29 Leninsky Avenue, Moscow119991, Russian Federation

**Keywords:** polymer of intrinsic microporosity, crosslinking, pervaporation, organic−organic separation, branched PIMs

## Abstract

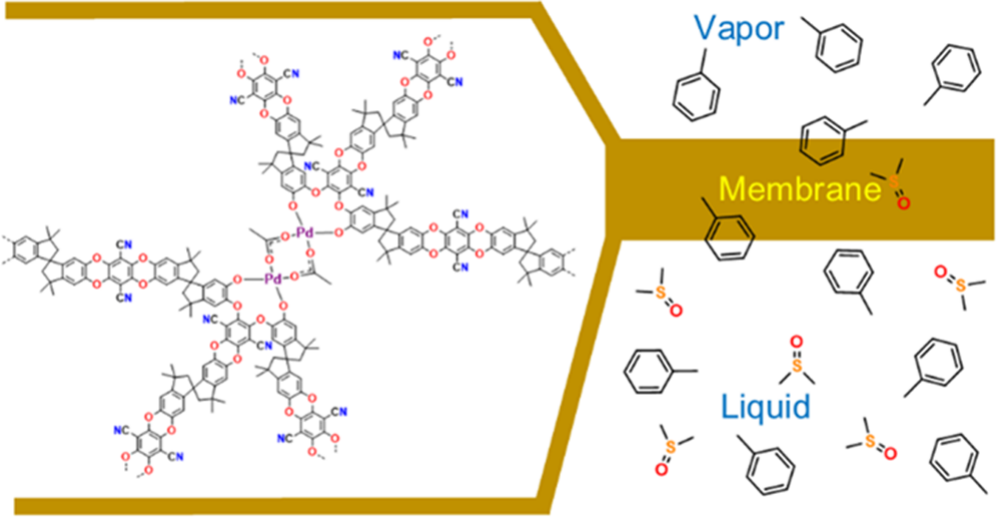

Branched forms of the archetypal polymer of intrinsic microporosity PIM-1 and the pyridinecarbonitrile-containing PIM-Py may be crosslinked under ambient conditions by palladium(II) acetate. Branched PIM-1 can arise in polymerizations of 5,5′,6,6′-tetrahydroxy-3,3,3′,3′-tetramethyl-1,1′-spirobisindane with tetrafluoroterephthalonitrile conducted at a high set temperature (160 °C) under conditions, such as high dilution, that lead to a lower-temperature profile over the course of the reaction. Membranes of PIM-1 and PIM-Py crosslinked with palladium acetate are sufficiently stable in organic solvents for use in the recovery of toluene from its mixture with dimethyl sulfoxide (DMSO) by pervaporation at 65 °C. With both PIM-1 and PIM-Py membranes, pervaporation gives high toluene/DMSO separation factors (around 10 with a 77 vol % toluene feed). Detailed analysis shows that the membranes themselves are slightly selective for DMSO and it is the high driving force for toluene evaporation that drives the separation.

## 1 Introduction

**Figure 1:**
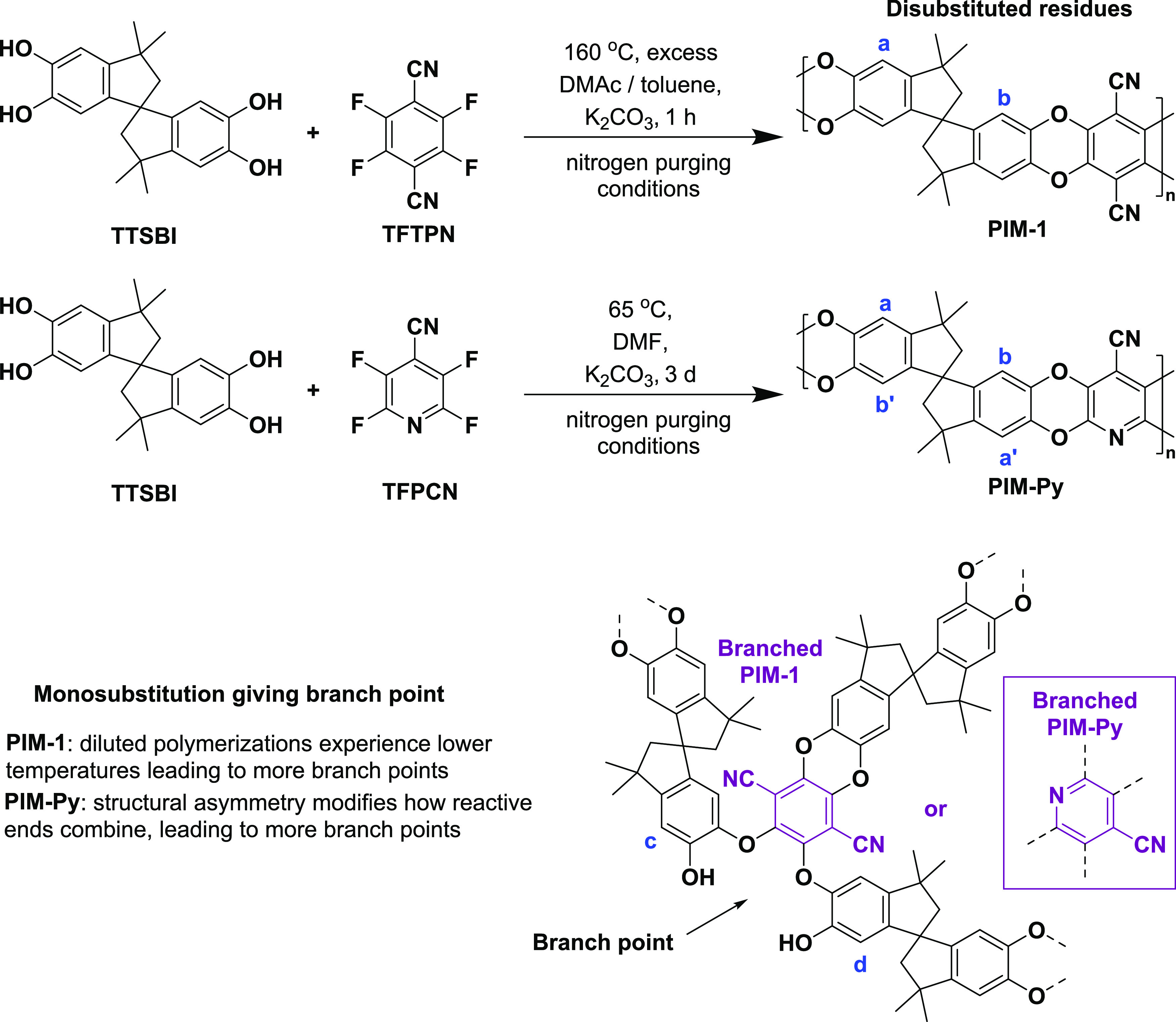
Syntheses of PIM-1 from 5,5′,6,6′-tetrahydroxy-3,3,3′,3′-tetramethyl-1,1′-spirobisindane (TTSBI) and tetrafluoroterephthalonitrile (TFTPN), and of PIM-Py from TTSBI and 2,3,5,6-tetrafluoro-4-pyridinecarbonitrile (TFPCN), showing ideal disubstituted structures and branched structures that may form as a result of monosubstitution.

Polymers of intrinsic microporosity (PIMs)^1,2^ are solution-processable, membrane-forming polymers that behave like microporous materials as defined by IUPAC (pore size <2 nm).^3^ Their polymeric backbones have many bends arising from sites of contortion such as spiro-centers, but their conformational freedom is limited because they lack single bonds in the backbone about which rotation can occur. In the glassy state, they possess a high degree of interconnected free volume, giving high permeability when used in membranes for gas separation^4^ or pervaporation (PV).^5,6^ It has been shown for the prototypical PIM-1^7,8^ and the pyridinecarbonitrile-containing PIM-Py (Figure 1)^9^ that variations in the polymerization conditions give products with different topologies, leading to different permeation properties. The present work has three objectives: (1) To establish polymerization conditions that produce high-molar-mass PIM-1 with a high degree of branching; (2) to show that films formed from strongly branched versions of PIM-1 and PIM-Py can be crosslinked by palladium(II) acetate; and (3) to demonstrate the application of the crosslinked films in the pervaporative separation of mixtures of toluene and dimethyl sulfoxide (DMSO).

Membranes of PIM-1 or PIM-Py may swell excessively or disintegrate when used with organic solvents or strongly adsorbing vapors. A variety of methods have been utilized to crosslink PIM membranes to stabilize them. Fritsch et al.^10^ produced thin-film composite membranes for organic solvent nanofiltration (OSN) from blends of PIMs with polyethyleneimine (PEI) and showed that a simple heat treatment of the PIM/PEI blend stabilized the membrane. Liao et al.^11^ and Zhao et al.^12,13^ first modified PIM-1 chemically, hydrolyzing it to introduce carboxylate functionality, then crosslinking it by metal ions. Putintseva et al.^14^ investigated these approaches to improve the stability of PIM-1 in mixtures of aromatic hydrocarbons with polar solvents. They found hydrolysis of PIM-1 followed by crosslinking with AlCl_3_ to be highly effective in studies of the separation of mixtures of toluene and triethylene glycol by thermopervaporation^15^ and of nanofiltration with a range of aromatic hydrocarbons and polar solvents.^16^ Crosslinking agents may be used with PIMs. Du et al.^17^ utilized diazide crosslinkers with PIM-1, forming crosslinks by a nitrene reaction at 175 °C under vacuum. A similar approach was taken by Khan et al.^18^ who employed poly(ethylene glycol) biazide as a crosslinker, undertaking the nitrene reaction at 250 °C. Tien-Binh et al.^19,20^ have reported crosslinking PIM-1 using metal–organic frameworks (MOFs) such as MOF-74 and UiO-66-NH_2_. Methane sulfonic acid (MSA) has been used to both modify and crosslink PIM-1 in a one-step method.^21^ Recently, it has been suggested that silicalite-1 undergoes noncovalent crosslinking with PIM-1 through π–π stacking interactions.^22^

There are various ways in which thermal treatment can bring about crosslinking of PIM-based membranes. Du et al.^23^ subjected PIM-1 membranes to base-catalyzed hydrolysis for varying times and thermally crosslinked them at 375 °C under an inert atmosphere. They suggested that crosslinking occurs via a decarboxylation mechanism. It should be noted, however, that base-induced hydrolysis may yield predominately amide rather than carboxylic acid functionality.^24^ Li et al.^25^ demonstrated crosslinking of unmodified PIM-1 membranes at temperatures in the range of 250–300 °C in a vacuum furnace. Song et al.^26^ showed that trace amounts of oxygen are critical for tuning the micropore structure when PIM-1 membranes undergo thermo-oxidative crosslinking at 385 °C. Chen et al. carried out thermal crosslinking of a bromomethylated PIM^27^ and blends of bromomethylated PIM with a Tröger’s base PIM^28^ at temperatures in the range of 200–300 °C. Higher-temperature treatments of PIMs lead to carbon molecular sieve membranes.^29^

Thermal treatments can stabilize membranes and enhance their separation properties, but they also introduce an energy-intensive processing step and tend to embrittle the membranes. There is a need for ways of crosslinking membranes post-fabrication under ambient conditions. McDonald et al.^30^ adsorbed the polycyclic aromatic hydrocarbons (PAHs) pyrene and 1-aminopyrene into PIM-1 films from methanolic solution, which brought about crosslinking through physical interactions between PIM-1 units and PAHs. In the present work, we demonstrate crosslinking with the transition-metal compound palladium(II) acetate.

Global production of aromatic hydrocarbons (arenes) referred to as BTX (benzene, toluene, and xylenes) exceeds 100 MT per year. In addition to being commonly used industrial solvents, they are part of the chain of feedstocks used in the production of many plastics, pharmaceuticals, dyes, detergents, insecticides, adhesives, and paints. Catalytic reforming of naphtha in a petroleum refinery yields BTX mixtures, which require further separation by a process such as extractive distillation. Polar solvents such as diethylene glycol, triethylene glycol, sulfolane, *N*-methyl-pyrrolidone, or DMSO are added as part of this process. A steam-stripping step is generally used to remove the arenes from the polar solvent, and there is interest in replacing this with a more energy-efficient membrane process. Solvent exchange processes are also important within pharmaceutical synthesis, and these often require separation of an aprotic solvent from a range of other solvents, including arenes.^31^ In the present work, the toluene/DMSO system is investigated as an example of the separation of an arene from an industrially important polar solvent.

Organic solvent-resistant membranes can be applied in a range of membrane processes, including OSN, organic solvent reverse osmosis (OSRO) or forward osmosis (OSFO), and PV.^32^ In PV, a liquid feed mixture is in direct contact with one side of the membrane and permeate is removed from the other side as a vapor. The first membrane application to be suggested for PIM-1 was the removal by PV of phenol from aqueous solution.^2^ PV membranes based on PIM-1 have been explored for recovery of ethanol^33,34^ and butanol^35−38^ from aqueous solution, and for the separation of methanol/dimethyl carbonate mixtures.^39^ Various organic–organic separations have also been reported for the high-free-volume material Teflon AF2400, recording relatively low fluxes (<1.7 kg m^–2^ h^–1^) and separation factors (<3.1).^40,41^ Membranes based on a spirobifluorene-PIM (PIM-SBF) have been investigated for PV of toluene/DMSO mixtures.^42^ In the present work, crosslinked membranes based on PIM-1 and PIM-Py are applied to this system and the results are analyzed in terms of permeance and selectivity.

## 2 Experimental Section

### 2.1 Polymer Synthesis

The chemicals and purification techniques employed are outlined in Section S1. Seven samples of PIM-1, derived from TTSBI and TFTPN, and one sample of PIM-Py, derived from TTSBI and TFPCN, are discussed in this work. The polymerizations are summarized in Table S1. Full details of the synthesis of two samples of PIM-1 (**6** and **7**) and of PIM-Py (**8**) are provided below.

#### 2.1.1 Synthesis of Branched PIM-1 (**6**)

Equimolar amounts of the monomers, TFTPN (100%, 10.00 g, 0.05 mol) and TTSBI (100%, 17.02 g, 0.05 mol) were placed along with potassium carbonate (20.73 g, 0.15 mol) into a 500 mL three-neck, round-bottom flask. The solvent mixture of dimethylacetamide (DMAc, 120 mL) and toluene (60 mL) was then added. This equates to an initial 20 vol % excess of solvent at the start of the reaction compared to the conventionally reported synthesis conditions.^7,43^ Both the larger scale of the reaction and the presence of extra solvent mean that the early stages of the polymerization proceed at a lower-temperature profile, which encourages more mono-substituted oligomeric structures within the mixture. The flask was equipped with a nitrogen inlet, coil condenser and a Heidolph mechanical stirrer (complete with digital rpm and torque reading display) which was used to mix the reaction mixture. A strong positive pressure of nitrogen was maintained over the reaction mixture. Heating was supplied via a hotplate equipped with a DrySyn aluminum heating block, with the flask inserted together with a temperature probe. The reaction mixture was heated from room temperature to 160 °C, initially stirred at 250 rpm, with the stirring rate increased or decreased as appropriate with changing viscosity of the overall mixture and torque readings recorded at regular intervals. After 27 min, an extra batch of DMAc (20 mL) and toluene (10 mL) solvent mixture was added to the flask to reduce the viscosity of the reaction mixture. This solvent batch addition was repeated after 36 min. A yellow solid visibly crashed out of solution at *ca.* 45 min into the reaction. After 60 min, the reaction mixture was quenched into excess methanol. The polymer yield obtained after purification (see below) was 22.5 g (98%). Molecular weight, *M*_w_ = 187 600, *M*_n_ = 90 000 (*Đ* = 2.1); ^1^H NMR (500 MHz, CDCl_3_, ppm) δ: 7.58 ppm hydroxyl proton, 6.81, 6.67, 6.42, 6.25 ppm aromatic protons (labeled **a**, **c**, **b**, **d** in Figure 1), 4.01, 3.93, 3.85 ppm hydroxyl protons, 2.29, 2.15 ppm methylene protons, 1.37, 1.31 ppm methyl protons, full ^1^H NMR spectrum presented in Figure S6; Elemental analysis (EA): C = 73.07%, N = 5.83%, H = 4.42%, F = 0.01%, predicted EA for disubstituted PIM-1 structure: C = 75.64%, H = 4.38%, N = 6.08%.; No measurable amount of colloidal network content was determined by filtration (<2.0%).

#### 2.1.2 Synthesis of Branched PIM-1 (**7**)

Polymerization **7** was also carried out on 50 mmol scale, with 3-fold excess of potassium carbonate and an initial 20 vol % excess of the DMAc/toluene solvent mixture. After 21 min, an additional 30 mL of the solvent mixture was added to the reaction based on increasing viscosity. This step was repeated 8 min later to further reduce the viscosity. The reaction was ended after only 36 min, with the reaction mixture poured into an excess of methanol. The polymer was filtered from the methanol and purified as described below. A polymer yield of 21.0 g (91%) was obtained. Molecular weight, *M*_w_ = 107 500 g mol^–1^, *M*_n_ = 56 600 (*Đ* = 1.9); ^1^H NMR (500 MHz, CDCl_3_, ppm) δ: 7.58 ppm hydroxyl proton, 6.81, 6.66, 6.42, 6.27 ppm aromatic protons (labeled **a**, **c**, **b**, **d** in Figure 1), 4.02, 3.93, 3.85 ppm hydroxyl protons, 2.33, 2.16 ppm methylene protons, 1.36, 1.31 ppm methyl protons; Elemental analysis (EA): C = 74.01%, N = 5.95%, H = 4.30%, F = 0.06%, predicted EA for disubstituted PIM-1 structure: C = 75.64%, H = 4.38%, N = 6.08%. Network content determined by filtration: 5%.

#### 2.1.3 Synthesis of Branched PIM-Py (**8**)

Equimolar amounts of the monomers, TFPCN (99.3%, 26.60 g, 0.15 mol) and TTSBI (100%, 51.06 g, 0.15 mol) were placed along with potassium carbonate (62.19 g, 0.45 mol) into a 2 L three-neck, round-bottom flask. The solvent, dimethylformamide (DMF) (600 mL) was then added. The flask was equipped with a nitrogen inlet, coil condenser, and a large PTFE magnetic stirring bar, which was used to mix the reaction mixture. A positive pressure of nitrogen was maintained over the reaction mixture. Heating was supplied via a hotplate equipped with a DrySyn aluminum heating block, with a temperature probe initially directly inserted into the reaction mixture. The reaction mixture was heated from room temperature to 65 °C. Once the internal temperature had stabilized at 65 °C, the temperature probe was removed, and the hotplate heating maintained at the level attained for 3 days. The mixture remained relatively low in viscosity throughout the reaction, with the hot reaction mixture then quenched into excess methanol after 3 days, precipitating as a yellow-green, thread-like PIM-Py polymer. The yield obtained after purification was 63.5 g (97%). Molecular weight, *M*_w_ = 223 200, *M*_n_ = 33 200 (*Đ* = 6.7); ^1^H NMR (500 MHz, CDCl_3_, ppm) δ: 7.72, 7.66, 7.59 ppm hydroxyl protons, 6.79, 6.73, 6.42, 6.37 ppm main resolved aromatic protons (labeled **a**, **a′**, **b**, **b′** in Figure 1), 4.02, 3.94, 3.85 ppm hydroxyl protons, 2.31, 2.15 ppm methylene protons, 1.34, 1.30 ppm methyl protons in the polymeric structure, full assigned ^1^H NMR spectrum presented in Figure S7; Elemental analysis (EA): C = 73.01%, H = 4.62%, N = 6.29%, predicted EA for disubstituted PIM-Py structure: C = 74.3%, H = 4.62%, N = 6.42%. Network content determined by filtration: 8%.

Each PIM polymer was purified as described in Section S2. The polymers were characterized as outlined in Section S3.

### 2.2 Membrane Fabrication and Crosslinking

#### 2.2.1 Initial Studies of Solid-State Crosslinking of PIM-1 Membrane with Pd(OAc)_2_

Films of 40 μm thickness were prepared from branched PIM-1 sample **6** as follows: A 3% w/v solution of the polymer in chloroform (∼0.12 g of the polymer in 4 mL of CHCl_3_) was cast into a PTFE Petri dish (diameter = 6 cm) and left for 4 days at room temperature in a positive nitrogen atmosphere cabinet to allow the film to slowly form and dry. The films self-detached from the PTFE surface of the dish over this period of time. The film thicknesses were confirmed with a Mitutoyo digimatic micrometer.

For crosslinking the membrane in the solid state, a known mass of PIM-1 **6** membrane (0.133 g, 0.289 mmol) was added to a large glass Petri dish (diameter = 10 cm) with Pd(OAc)_2_ (0.0648 g, 0.289 mmol) which equates to 100 mol % of metal salt per PIM-1 residue present in the mass of the film. Methanol (30 mL) was added to the dish with a stirrer bar, the contents were covered with another inverted Petri dish and left to mix for 4 h. Pd(OAc)_2_ is only minimally soluble in methanol and the majority of the metal salt remains clearly visible in the methanol. After this period, the solution and film exhibited a dark brown color. The methanol supernatant was then removed, and the film was washed with more methanol (30 mL). Some of the initially added Pd(OAc)_2_ had remained insoluble in methanol in the dish. This process was repeated twice, with the methanol supernatant removed each time. The crosslinked film was placed in a N_2_ cabinet to dry overnight. The final film displayed a dark yellow/brown color. The film was repeatedly washed in consecutive volumes of chloroform (3 × 30 mL) to determine the relative success of crosslinking. The film expanded in diameter (from 5.0 to 8.6 cm) immediately on immersion in the solvent, without the chloroform solution taking on significant color (visual evidence of a distinct lack of PIM-1 polymer leaching from the film). The collected chloroform washings concentrated to a green solid, which when redissolved in THF at a known concentration (0.09 mM) for UV–vis absorption spectroscopy analysis confirmed negligible PIM-1 content. The film was then dried in a N_2_ cabinet overnight and reweighed, indicating a mass increase of 3.9%. If this mass difference is entirely attributable to Pd(OAc)_2_ within the film, this equates to a film containing 1.8 wt % of palladium (Pd). Elemental analysis indicated a Pd content of 2.2 wt % in the crosslinked film (first entry denoted as *x*PIM-1 **6** in Table S5). This indicated that only 11–14 mol % of the palladium acetate was required to fully crosslink the branched PIM-1 film. The thickness of the enlarged crosslinked film proved to be only 20 μm.

A thicker initial film of PIM-1 polymer **6** (60 μm), crosslinked in the solid state with 25 mol % of Pd(OAc)_2_, similarly enlarged in diameter on post-treatment in chloroform and contracted in thickness to 42 μm (Table S5). Elemental analysis indicated that this crosslinked film contained 1.71 wt % of Pd.

#### 2.2.2 Solid-State Crosslinking of PIM-1 and PIM-Py Membranes with Pd(OAc)_2_ for Pervaporation Studies

Films of branched PIM-1 **6** and **7** with thicknesses in the range 60–100 μm and of PIM-Py **8** with thicknesses in the range 38–77 μm were crosslinked and used for pervaporation studies of toluene/DMSO mixtures. A typical procedure for PIM-1 **7** is as follows: A solution of the polymer in chloroform (∼0.20 g of the polymer in 10.0 mL of CHCl_3_) was cast into a PTFE Petri dish (diameter of 6 cm) and left for 4 days at room temperature in a positive nitrogen atmosphere cabinet to allow the film to slowly form and dry. The weight of each pristine dry film (for example, 0.200 g, 0.434 mmol) was determined and used to calculate 25 mol % of Pd(OAc)_2_ equivalence addition (0.0243 g, 0.108 mmol), a slight excess on the 11–14 mol % of Pd(OAc)_2_ shown to be sufficient to crosslink a branched PIM-1 film. The film and Pd(OAc)_2_ were transferred to a large glass Petri dish and immersed in methanol (30 mL) for 4 h. The methanol was decanted and the film was washed a couple of times in methanol (2 × 30 mL) before the dark yellow/brown film was left to slowly dry in a N_2_ cabinet overnight. Each film was then repeatedly washed in consecutive volumes of chloroform (3 × 30 mL). The films again expanded in diameter immediately on immersion in the solvent. Some of this lower-molar-mass PIM-1 (**7**) polymer dissolved into the chloroform washings, but the films remained intact. The final dried films exhibited a significant drop in thickness to 20–30 μm as a consequence of the treatment.

It should be noted that when solid-state crosslinking was carried on even thicker (80–100 μm) self-standing films of PIM-1 **7**, while they remained intact on washing repeatedly with chloroform, sometimes considerably more polymer was dissolved in the chloroform washings. Multiple washings (5 × 30 mL) with chloroform were often required until the solutions were almost colorless. The final crosslinked films exhibited reduced thicknesses of 20–80 μm and were also used in some pervaporation experiments.

Branched PIM-Py (**8**) films were treated with 25 mol % of Pd(OAc)_2_ for 4 h in the same manner. Enlarged crosslinked films of reduced thickness, stable in chloroform, were prepared and used in pervaporation studies. Elemental analysis indicated levels of Pd of 1.4–2.2 wt % within the crosslinked films (denoted as *x*PIM-Py **8** in Table S5).

### 2.3 Pervaporation

**Figure 2:**
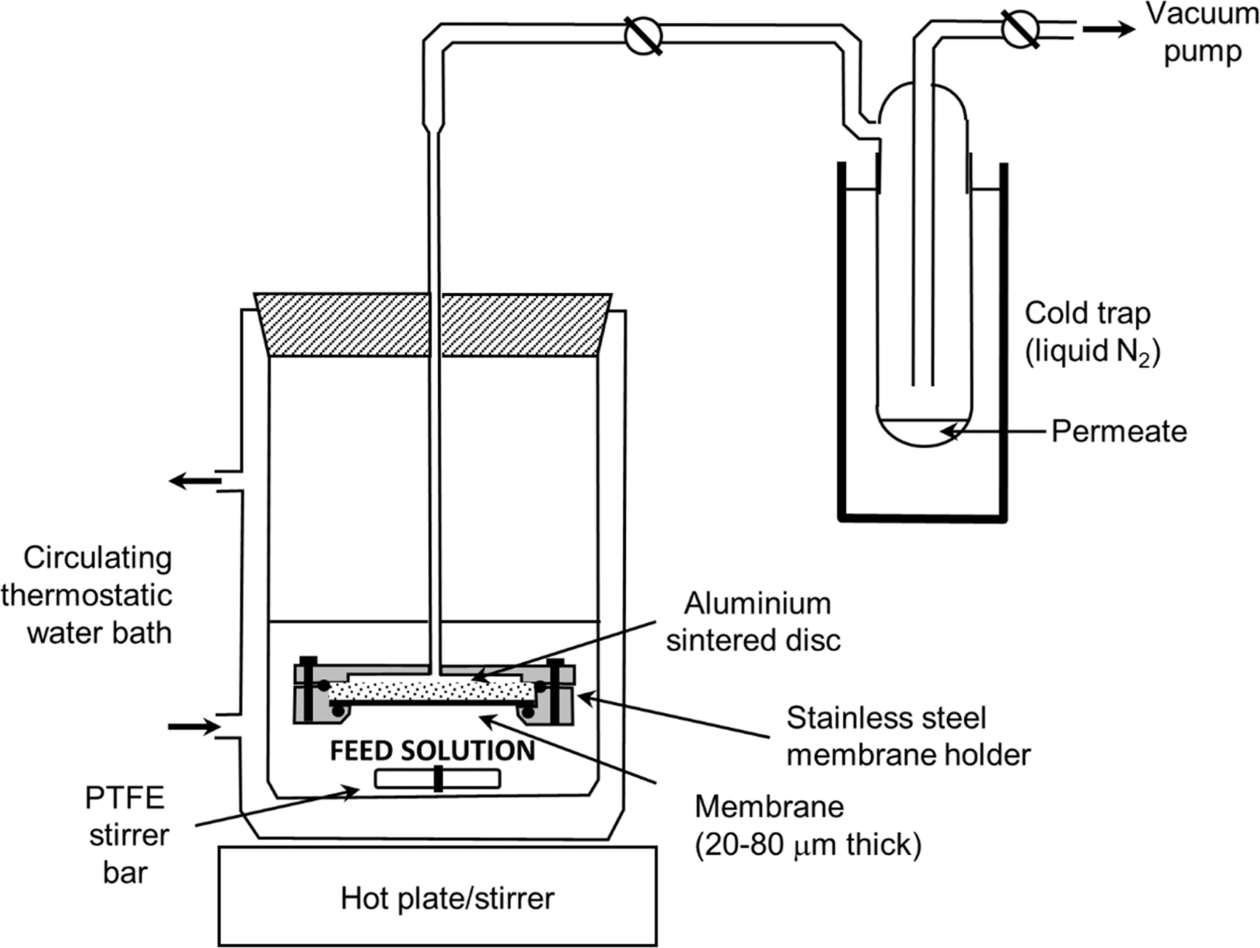
Batch pervaporation setup used to separate toluene/dimethyl sulfoxide feed mixtures.

A batch PV apparatus was utilized in which vacuum is applied on the permeate side to generate a driving force for permeation, and the permeate is then condensed and collected in a liquid nitrogen cold trap (Figure 2). An Edwards E2M18 vacuum pump was used to maintain a low pressure (10 mbar) on the permeate side during the experiment. An O-ring with an internal diameter of 1.95 cm was used to seal the membrane in the housing, giving an active membrane area of 2.99 cm^2^. A Viton O-ring was a prerequisite as a nitrile O-ring failed with organic solvents. A double-jacketed glass vessel was connected to a digital thermostatic heating water bath to stabilize the temperature on the feed side at 65 °C during PV. Experiments were conducted with an initial feed volume of 40 cm^3^ and a range of initial feed compositions (87:13, 77:23, 60:40, 50:50, 40:60, 30:70, 20:80, and 10:90 toluene/DMSO by volume). In most cases, permeate was taken, weighed, and analyzed by gas chromatography (see below) after 2, 4, and 6 h, but at low toluene concentrations PV was allowed to proceed for a longer time period to collect sufficient permeate for analysis. For each initial feed composition, up to four separate runs were carried out with fresh membranes.

#### 2.3.1 Gas Chromatography (GC) Analysis

Toluene and DMSO concentrations were determined by GC analysis. Each permeate or feed sample of toluene–DMSO tested was diluted with dichloromethane (DCM) to reach the established calibration range for the two solvents. Analysis was performed using an Agilent 6890 gas chromatography system with a flame ionization detector, which responds to toluene, DMSO, and DCM. The concentrations of toluene and DMSO in the diluted sample were determined from the respective areas of the toluene and DMSO peaks using calibrations from measurements of toluene/DMSO/DCM mixtures of known composition. The composition of the original sample was then calculated based on the known dilution. The column was a Zebron ZB Semi-Volatiles (part number 7HG-G027-11GGA) 30 m × 0.25 mm, ID 0.25 μm film thickness. Helium was used as carrier gas with a constant flow rate of 1.0 mL min^–1^. The ion source was set at a temperature of 300 °C. The oven temperature programming was initially 70 °C, kept isothermal for 5 min, ramped at 10 °C min^–1^ up to 250 °C, then at 25 °C min^–1^ up to 300 °C and isothermal again at this temperature for 3 min (total run time of 28 min). The injection volume was 1 μL, applying a split ratio of 20:1. The toluene and DMSO peaks appear after scanning times of 4.3 and 5.3 min, respectively.

#### 2.3.2 Analysis of Pervaporation Data

Total mass flux was calculated using
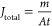
1where *m* is the total mass of permeate collected over time *t* for a membrane of area *A*. Separation factor was calculated using
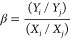
2where *Y*_*i*_/*Y*_*j*_ is the weight or molar ratio of the components in the permeate and *X*_*i*_/*X*_*j*_ is the corresponding ratio in the feed. In this work, the permeate is enriched in toluene, which is taken as component *i*, and DMSO is component *j*. As an indication of the overall performance, pervaporation separation index (PSI) was calculated using
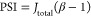
3

The mass flux, *J*_*i*_, of component *i* was calculated using
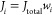
4where *w*_*i*_ is the weight-fraction of component *i* in the permeate. The molar flux, *j*_*i*_, of component *i* was calculated using
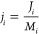
5where *M*_*i*_ is the molar mass of component *i*. This was multiplied by the molar volume of an ideal gas at standard temperature and pressure (22.4 L at STP) to convert the amount in moles to an equivalent volume. To evaluate permeance, permeability, and selectivity, the driving force was calculated as
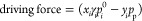
6where *x*_*i*_ is the mole fraction, γ_*i*_ is the activity coefficient and *p*_*i*_^0^ is the vapor pressure of component *i* on the feed side, *y*_*i*_ is the mole fraction of component *i* on the permeate side, and *p*_p_ is the total pressure on the permeate side. Activity coefficients and vapor mole fractions for toluene/DMSO mixtures at 65 °C were calculated using ASPEN PLUS (version 11) simulation software with the NRTL thermodynamic model. Values of permeability (*P*_*i*_) and permeance (*P*_*i*_/*l*) were calculated from molar flux using
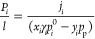
7where *l* is the membrane thickness. As is common in the membrane literature, permeability is expressed in units of barrer (1 barrer = 10^–10^ cm^3^ [STP] cm cm^–2^ s^–1^ cmHg^–1^ = 3.35 × 10^–16^ mol m m^–2^ s^–1^ Pa^–1^) and permeance in gas permeation units (1 GPU = 10^–6^ cm^3^ [STP] cm^–2^ s^–1^ cmHg^–1^ = 3.348 × 10^–10^ mol m^–2^ s^–1^ Pa^–1^). It is worth noting that permeability in barrer is obtained by multiplying permeance in GPU by thickness in μm. The membrane selectivity is expressed as a ratio of permeances or permeabilities.
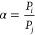
8

## 3 Results and Discussion

### 3.1 Synthesis and Characterization of Branched PIMs

Previous work on the effect of polymerization conditions on the topology of PIM-1 prepared from TTSBI and the chloro-monomer tetrachloroterephthalonitrile^7,8^ indicated that monosubstitution rather than disubstitution may occur at intermediate polymerization temperatures (80–140 °C), leading to branched structures. Historically, such branched structures have also arisen in PIM-1 formed in some polymerizations of TTSBI with the fluoro-monomer TFTPN in DMAc/toluene mixtures at a high set temperature (160 °C) (polymers **1** and **2** discussed in Section S4) and this is explored in the present work. Polymerizations of TTSBI with TFPCN to yield PIM-Py are particularly prone to produce branched structures^9^ and a branched sample of PIM-Py was also prepared in the present work.

**Figure 3:**
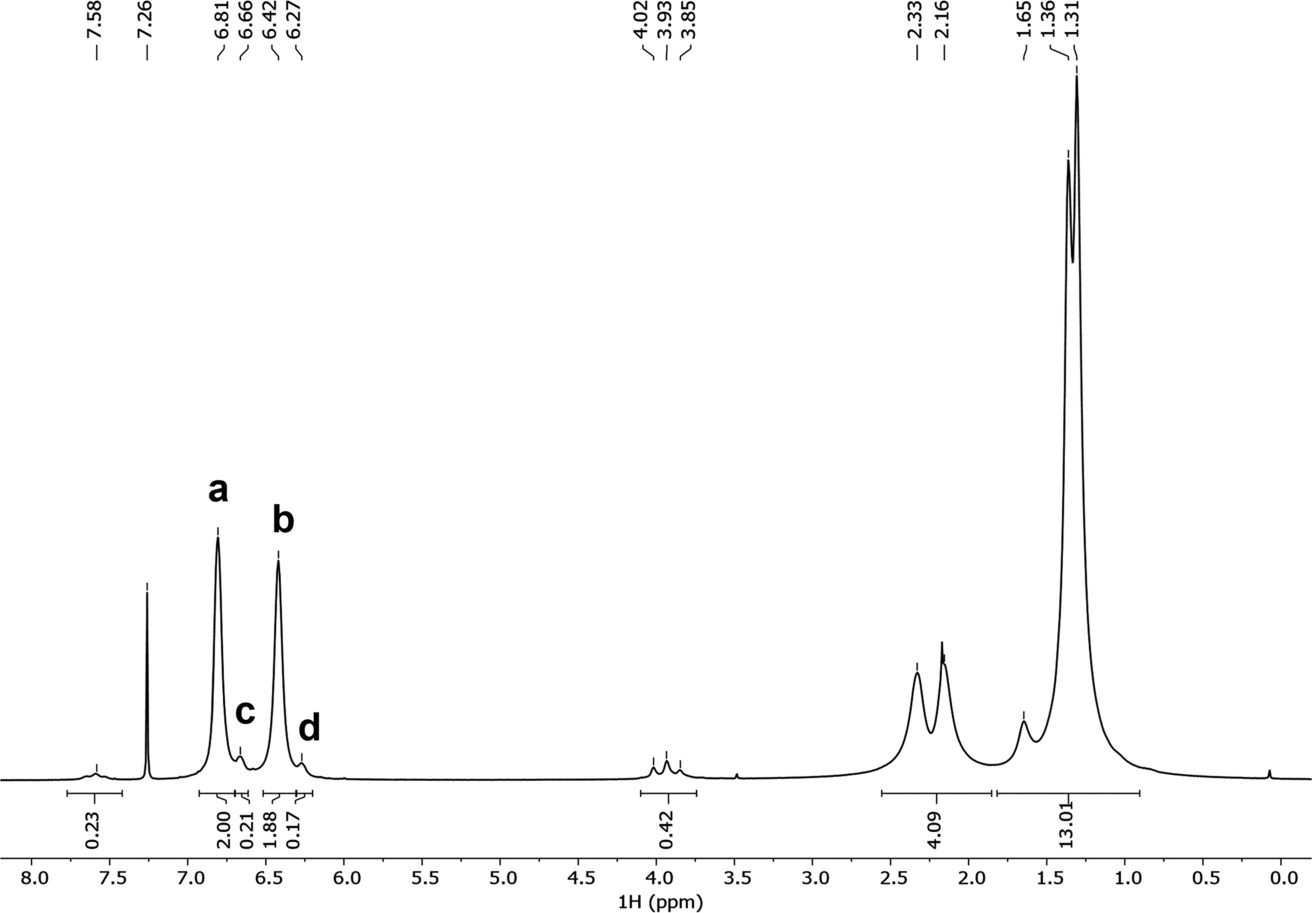
^1^H NMR spectrum of branched PIM-1 polymer **7** (*M*_w_ = 107 500, *Đ* = 1.9), which was used in membranes and crosslinked with Pd(OAc)_2_ for pervaporation experiments. For assignments of peaks **a**, **b**, **c**, and **d**, see Figure 1.

The primary evidence for branched structures comes from the aromatic region of the ^1^H NMR spectra. The ^1^H NMR spectrum of PIM-1 sample **7** is shown in Figure 3, and spectra for other samples are in Supporting Information (Figures S1–S7). In CDCl_3_, PIM-1 with the idealized, disubstituted structure exhibits two main aromatic resonances (**a** and **b**) at around 6.81 and 6.42 ppm. Defects associated with branches give rise to a minor peak upfield of each main peak (**c** and **d**), at around 6.66 and 6.27 ppm.^7^ In PIM-Py, there are four main aromatic peaks (**a**, **a′**, **b** and **b′**), at 6,79, 6.73, 6.42, and 6.36 ppm (Figure S7), because of the asymmetry of the neighboring pyridine residue, and defects associated with branches give rise to minor peaks (**c** and **d**) at 6.66 and 6.27 ppm.^9^ The amount of branching as a percentage of all PIM residues present was calculated from the relative sizes of the minor peaks expressed as % area relative to major peaks following deconvolution (see Supporting Information Section S3 and Tables S3 and S4).

Five PIM-1 polymerizations of TTSBI with TFTPN (polymers **3**–**7**, Table S1) are discussed here. Characterization data for these PIM-1 samples (**3**–**7**), and for PIM-Py (**8**), are tabulated in Table 1. Apart from **5**, the PIM-1 polymerizations were carried out in a DMAc/toluene mixture (2:1 by volume) with 3-fold molar excess of potassium carbonate at a set temperature of 160 °C. However, the actual temperature profile of the reaction mixture over the course of the polymerization depends on several factors. Average temperatures are indicated in Table S1 where data are available.

**Table 1:** PIM-1 and PIM-Py Characterization Data

	^1^H NMR	multidetector SEC				
polymer	branching (%)^a^	*M*_w_(kg mol^–1^)	*Đ*	[η] (cm^3^ g^–1^)	*K*(cm^3^ g^–1^)^b^	*a*^b^
PIM-1**3**	3.7	116.3	2.0	35.0	0.0182	0.656
PIM-1**4**	6.5	142.6	2.5	39.3	0.0140	0.679
PIM-1**5**	<2.0^c^	119.2	2.2	29.1	0.0137	0.664
PIM-1**6**	17.0	187.6	2.1	46.6	0.0063	0.743
PIM-1**7**	10.5	107.5	1.9	33.8	0.0204	0.652
PIM-Py**8**	26.0	223.2	6.7	35.4	0.1113	0.492

a Defect peaks attributed to branch points compared as percentage of major peaks attributed to disubstituted PIM residue structures as outlined in Tables S3 and S4. PIM-1 polymer samples, **3**–**6**, exhibited very low levels of network, not measurable by filtration (<2%), PIM-1 **7** and PIM-Py **8** polymer sample contained 5 and 8% network content, respectively.

b *K* and *a* parameters from linear fit to Mark–Houwink plot over the molar mass range 50 000–400 000 g mol^–1^.

c Defect peaks not clearly resolved from the major peaks. Branching estimation provided. Linear disubstituted PIM-1 sample (dashed line in Figure 4) exhibits Mark–Houwink parameters: *K* = 0.0196, *a* = 0.646.

A hypothesis explored in this work was that higher dilution at the start of the polymerization, coupled with further addition of solvent mixture toward the end of the polymerization to maintain uniform mixing, would yield a richly branched PIM-1 sample. A slightly lower-temperature profile should favor more mono-substituted linkages and the formation of a higher proportion of branched oligomers at the early stages of the step-growth polymerization. This point is illustrated in the structural differences observed in polymers produced by reactions **3** and **4**, synthesized using two very different-sized heating blocks, which meant that the flasks were heated from room temperature at different rates in otherwise identical polymerizations. The small heating block, which supplied heat equating to an average reaction temperature of 141 °C during the 30 min reaction, yielded a predominantly disubstituted PIM-1 sample, polymer **3** (Figure S3, only 3.7% branched). The much larger DrySyn Classic heating block provided an average reaction temperature of 127 °C during a 40 min reaction, producing a more strongly branched PIM-1 sample, polymer **4** (Figure S4, 6.5% branched). The large block was used in all other 0.05 mol scale reactions.

The role of the solvent mixture was also investigated. Polymerization **5** was carried out in a DMAc/dichlorobenzene (DCB) solvent mixture (2:1 by volume) at a set temperature of 140 °C. Polymerizations in DMAc/DCB mixtures maintain a more homogeneous composition throughout the course of the reaction, compared to polymerizations in DMAc/toluene mixtures, where the polymer tends partially to precipitate out of solution during the reaction. However, the products of polymerization in DMAc/DCB mixtures generally contain some colloidal network material (<100 nm in diameter). This most likely arises from secondary reactions at branch points that give rise to four-way linkages, as discussed previously by Foster et al.^8^ There is little evidence of the minor aromatic peaks in the ^1^H NMR spectrum of polymer **5** (Figures S5 and S8, <2.0% branched), suggesting that the non-network material is essentially disubstituted. High-molar-mass PIM-1 polymers **4**, **6**, and **7**, produced in DMAc/toluene, are significantly branched (6.5, 17.0, and 10.5%, respectively) but do not contain significant amounts of colloidal network material.

Information about the molar mass distribution and intrinsic viscosity of the polymer samples was obtained by triple detector SEC. Table 1 gives values of weight-average molar mass, *M*_w_, dispersity, *Đ* (=*M*_w_/*M*_n_, where *M*_n_ is number-average molar mass) and intrinsic viscosity, [η], in the solvent used for analysis (chloroform). Triple detector SEC also provides information about the dependence of [η] on molar mass, *M*, over the range of the sample’s molar mass distribution. Over a large part of the distribution, these data follow the Mark–Houwink relationship
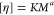
9where *K* and *a* are constants for a particular polymer in a particular solvent at a certain temperature. Mark–Houwink plots of log_10_[η] versus log_10_*M* for the PIM-1 samples **3**–**7** in chloroform at 35 °C are presented in Figure 4, and values of *K* and *a* over the molar mass range 50 000–400 000 g mol^–1^ are included in Table 1. The dashed line in Figure 4 indicates the relationship observed for linear, disubstituted PIM-1,^7^ for which *K* = 0.0196 cm^3^ g^–1^ and *a* = 0.646. For conventional, flexible polymers, branched molecules show a lower intrinsic viscosity than linear molecules of the same molar mass, and the slope of the Mark–Houwink plot is less for a branched polymer than for an equivalent linear polymer.^44^ However, the richly branched PIM-1 samples **4**, **6**, and **7** behave differently to flexible branched polymers, with Mark–Houwink plots that are similar to, or even above that of, linear disubstituted PIM-1, over much of the molar mass range. This suggests that the inflexible backbone maintains a relatively open structure for branched PIM-1. The branched PIM-Py sample **8**, exhibits a lower *a* value of 0.492 over the molar mass range considered.

**Figure 4:**
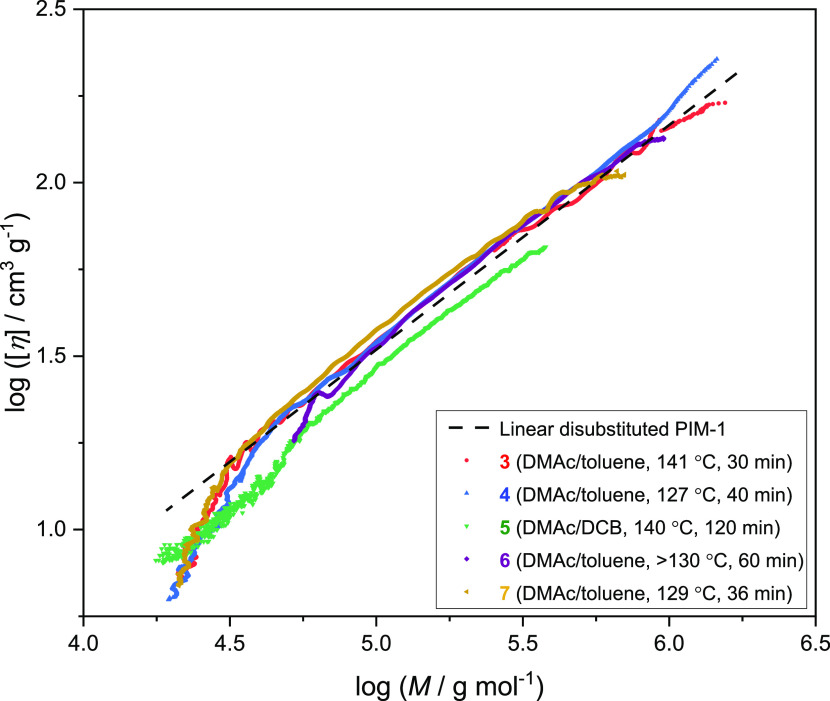
Mark–Houwink plots of branched PIM-1 samples **4**, **6**, and **7**, predominately disubstituted PIM-1 sample **3** and cyclic-rich PIM-1 sample **5**, in chloroform at 35 °C. The dashed line indicates the behavior expected for linear, disubstituted PIM-1.^7^

It can be seen in Figure 4 that PIM-1 sample **5**, believed to be predominately disubstituted as discussed above, has a Mark–Houwink plot that lies below that expected for linear PIM-1 over the whole molar mass range. This is indicative of a cyclic structure.^7^ A cyclic PIM has a more compact structure with lower intrinsic viscosity than a linear polymer of the same molar mass, as is also observed for a conventional, flexible polymer.^45^

### 3.2 Interactions of Branched PIM-1 with Palladium(II) Acetate in Chloroform Solution

Initial screening of topologically different PIM-1 polymer samples showed that high-molar-mass, branched PIM-1 in chloroform solution (3% w/v) interacts strongly with palladium (II) acetate (Pd(OAc)_2_) (when present at 50 mol % levels per PIM-1 residue), to the extent that a gel is formed, as illustrated in Figure S12a for PIM-1 sample **6**. Figure S12b compares the NMR region associated with aromatic protons for the whole PIM-1 **6** with that for the remnant that remained in solution after gelation. The polymer that is not incorporated into gel does not show the resonances at 6.7 and 6.3 ppm, previously assigned to defects associated with branches. This suggests that the gel is formed by crosslinking of PIM-1 chains by Pd(OAc)_2_ at the branch points. Less branched PIM-1 samples show some evidence of smaller colloidal structures forming in solution over time. No similar interaction in solution was observed with other metal salts, such as iron(II) acetate, palladium(II) chloride or iron(II) chloride.

**Figure 5:**
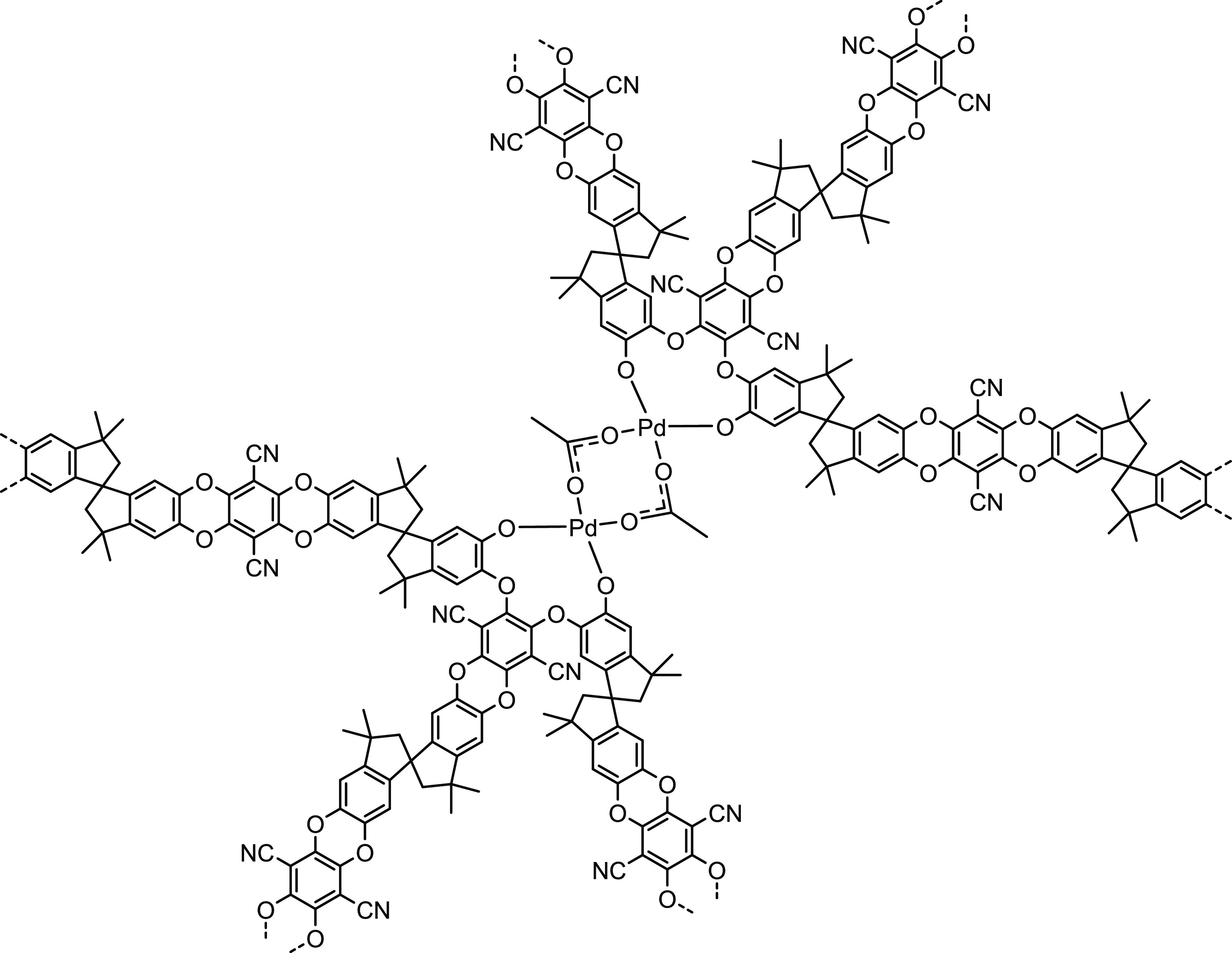
Possible interaction of Pd(OAc)_2_ with PIM-1 branch points to give crosslink.

Pd(OAc)_2_ is known to exist as a cyclic trimer in noncoordinating solvents such as toluene, and as a solvated monomer in coordinating solvents such as pyridine.^46^ It can be precipitated in a polymeric form.^47^ Stoyanov^48^ reported that for Pd(OAc)_2_ in chloroform solution there is an equilibrium between cyclic trimer, linear dimer, and monomer, with a shift toward dimer and then monomer on dilution. We tentatively suggest that in the presence of branched PIM-1, ligand exchange leads to crosslink structures such as that illustrated in Figure 5.

The rapid rate at which solutions of branched PIM-1 polymer in chloroform form a gel in the presence of Pd(OAc)_2_ means that it is not possible to maintain solvent miscibility long enough to cast a self-standing robust film directly from solution. If a film does form, it tends to be very brittle and not conducive to use in a membrane application. Therefore, a method was established to crosslink preformed polymer films as discussed below.

### 3.3 Solid-State Crosslinking of Membranes with Palladium(II) Acetate

**Figure 6:**
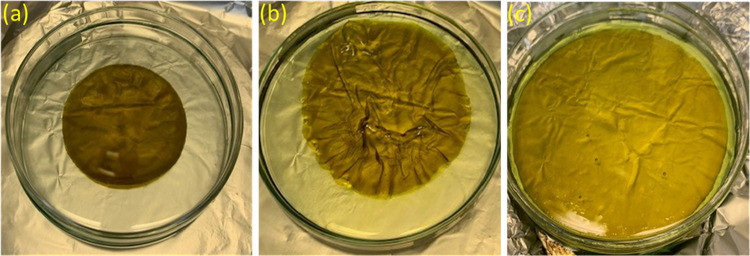
PIM-1 film (a) after treating with 100 mol % Pd(OAc)_2_ in methanol for 4 h followed by washing with methanol, (b) shown expanding in diameter upon initial immersion in chloroform and (c) fully expanded film after repeated washing in chloroform.

Crosslinking of membranes formed of branched PIMs was carried out by immersion of the membrane in methanol, which does not dissolve the membrane, in the presence of Pd(OAc)_2_. Although Pd(OAc)_2_ is only minimally soluble in methanol, there is sufficient transfer of Pd(OAc)_2_ into the membrane for crosslinking to occur. Initial crosslinking studies were carried out on 40 μm thick films of branched PIM-1 sample **6**, as described in Section 2. A 4 h treatment gave a film that did not dissolve in chloroform (a solvent for the uncrosslinked polymer), although the membrane did show an increase in diameter (Figure 6) and a reduction in thickness on immersion in chloroform. 11–14 mol % of the Pd(OAc)_2_ was required to fully crosslink the branched PIM-1 film. The original films were prepared in PTFE dishes, smaller than the glass petri dish shown in Figure 6a; there is no discernible change on initial crosslinking, other than change in film color. As shown in Figure 6b,c, the newly crosslinked PIM films actually expanded in overall volume (50–113% increase) after treatment with chloroform. UV–vis analysis of the solution indicated that the Pd(OAc)_2_-treated PIM films prepared from heavily branched polymers **6** and **8** lost very little PIM material during this expansion when treated with chloroform.

When the treatment time was extended from 4 to 24 h, both the solution and film turned black. After drying, the black film proved to be extremely brittle. The dark color is an indication of reduction of Pd(II) to Pd(0). There was also evidence that palladium was present on the surface of the film that required vigorous cleaning to remove. It was concluded that a 24 h treatment was excessive and a 4 h treatment was sufficient to obtain a crosslinked PIM-1 film.

It should be noted that much less polymer leached out in the chloroform washing stage from more highly branched PIM-1 polymers (PIM-1 **1** and PIM-1 **6**, 13.5 and 17.0% branched, respectively) in films crosslinked with Pd(OAc)_2_, compared, for example, to PIM-1 **7** (10.5% branched). For these particular branched polymers, this equates on average to between 5 and 9 PIM-1 residues per branch point. This work suggests that crosslinking is most effective with high-molar-mass polymers (*M*_w_ > 100 000 g mol^–1^) which are at least 10% branched.

The IR spectrum of the solid-state crosslinked PIM-1 film (**6**) is presented in Figure S12c. An extra peak present at 1603 cm^–1^ may be attributed to C=O asymmetric stretching of the carboxylate (−OCOCH_3_) groups^49^ still bound to palladium within the film. This confirms that intact Pd(OAc)_2_ remains coordinated within the film. Comparisons can be made to IR spectra obtained for a disubstituted PIM-1 polymer (**5**) and a more heavily liquid phase Pd(OAc)_2_ treated film (50 mol % Pd(OAc)_2_ present) from this polymer in Figures S13 and S14, respectively.

Treatment conditions and elemental analyses for membranes used in pervaporation studies are summarized in Table S5.

### 3.4 Pervaporation of Toluene/DMSO Mixtures

#### 3.4.1 Pervaporation of Toluene/DMSO Mixture (77:23 Volume Ratio) with Crosslinked PIM-1 and PIM-Py Membranes

**Figure 7:**
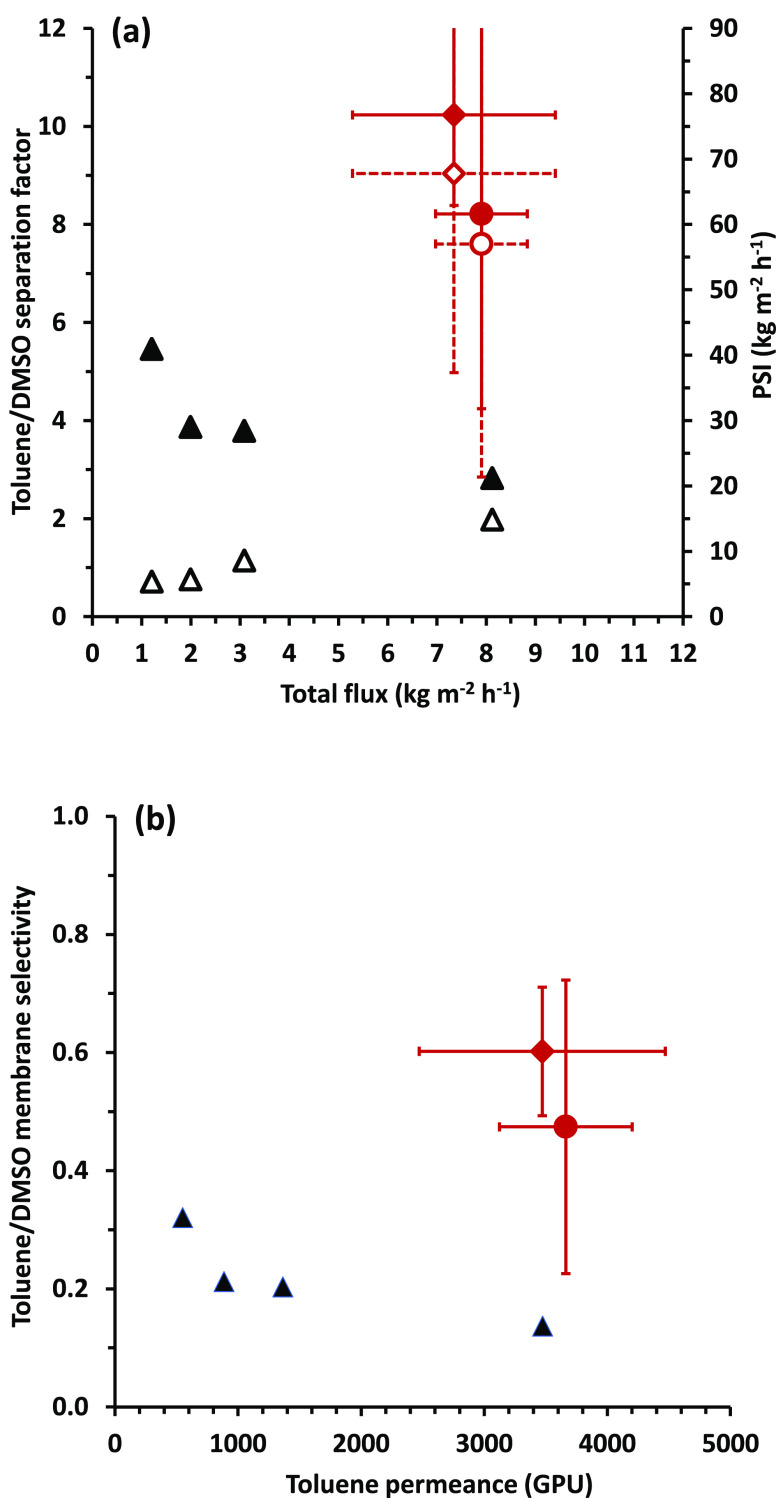
Pervaporation at 65 °C with a toluene/DMSO (initially 77:23 by volume) feed. Relationships between (a) toluene/DMSO separation factor (solid symbols, left-hand axis), PSI (open symbols, right-hand axis), and total flux and (b) toluene/DMSO membrane selectivity and permeance, for PIM-1 (red circle solid, red circle open) and PIM-Py (red diamond solid, red diamond open) crosslinked with Pd(OAc)_2_ (error bars indicate standard deviation for three experiments with different membranes), compared with data for membranes based on PIM-SBF (▲, Δ).^42^

Membranes formed of branched PIM-1 and PIM-Py, crosslinked with Pd(OAc)_2_, were used for pervaporation of a toluene/DMSO mixture with an initial feed composition of 77 vol % toluene at a temperature of 65 °C with a permeate pressure of 10 mbar. Figure 7a compares the performance of crosslinked PIM-1 and PIM-Py with previous data^42^ for PIM-SBF (with and without the addition of polyphenylene network fillers). Both PIM-1 and PIM-Py give higher separation factor and PSI than achieved in the previous work, together with a flux that is similar to that obtained before with the most permeable filler. The error bars in Figure 7a reflect some variation between different membranes of the same polymer. There are variations in the initial thicknesses of the membranes (see Tables S6 and S7) and the membranes may swell to differing extents under the conditions of use. Also, the concentrations of DMSO in the permeate are very low, making quantification difficult. For an average of three separate experiments with different membranes, PIM-Py gave slightly higher separation factor (10) and PSI (68 kg m^–2^ h^–1^) with slightly lower flux (7.4 kg m^–2^ h^–1^) than PIM-1. The fluxes achieved in pervaporation under the conditions used here are much higher than those obtained by Chau et al.^31^ for separation by reverse osmosis of a toluene/DMSO mixture of similar composition. These results illustrate the potential of PIM membranes, crosslinked post-fabrication, for organic/organic separations.

As pointed out by Wijmans,^50,51^ the performance of a membrane process depends both on the membrane properties and on the operating conditions. If we are to separate the role of the membrane from the effects of different operating conditions, we should calculate intrinsic membrane properties for each component, taking into account the driving force for permeation. In pervaporation, the driving force arises from the difference in partial pressure between the feed and permeate sides of the membrane.^52^

Permeance and selectivity were calculated using eqs 7 and 8 (see Section 2). Figure 7b shows how toluene/DMSO membrane selectivity relates to toluene permeance for PIM-1-, PIM-Py-, and PIM-SBF-based membranes. It is noteworthy that in all cases the membrane is actually selective for the more polar DMSO over toluene (toluene/DMSO membrane selectivity <1). Toluene (boiling point 111 °C) is favored over DMSO (boiling point 189 °C) in the permeate because of the large driving force for permeation arising from its higher volatility. Ironically, the high separation factors observed in Figure 7a for PIM-1 and PIM-Py arise because the membranes are less selective than with PIM-SBF. Pervaporation, as the name indicates, involves both permeation and evaporation, and in this case it is evaporation that dominates the observed separation. This is discussed further below.

#### 3.4.2 Pervaporation of Toluene/DMSO Mixtures over a Wide Composition Range with Crosslinked PIM-1 Membranes

**Figure 8:**
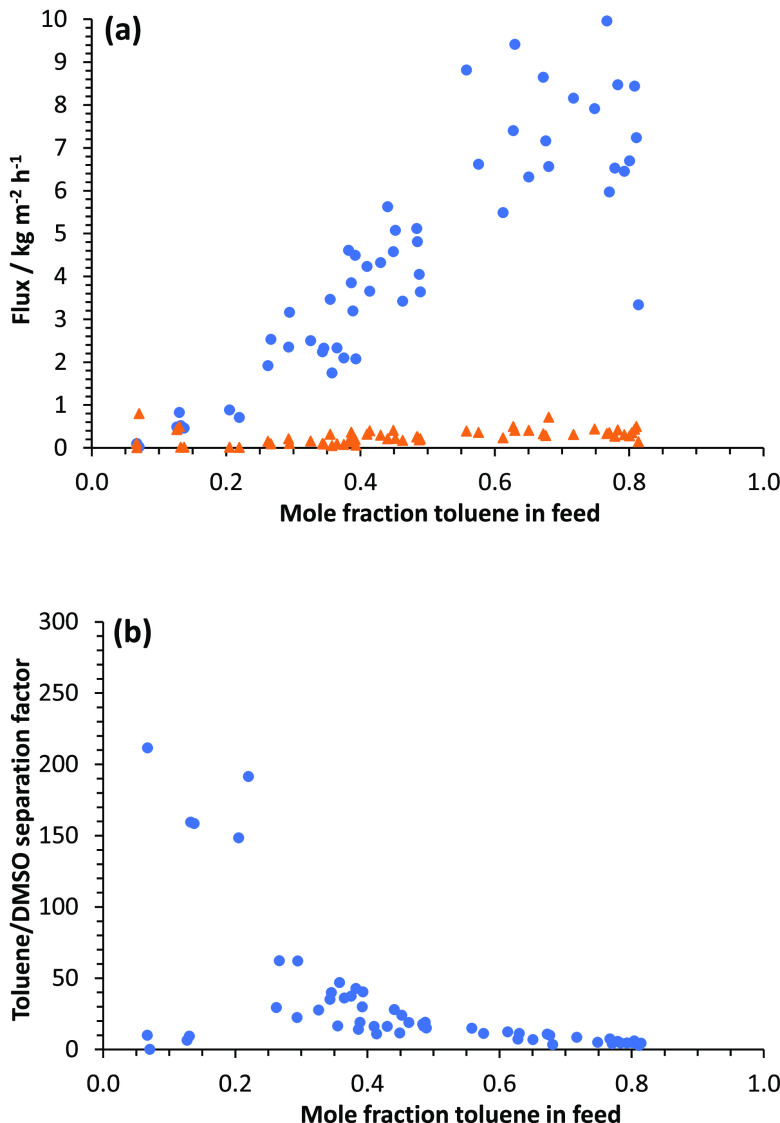
Dependence of (a) toluene flux (blue circle solid) and DMSO flux (orange triangle solid) and (b) toluene/DMSO separation factor on mole fraction of toluene in the feed (average over the time period of permeate collection) for pervaporation at 65 °C using branched PIM-1 membranes crosslinked with Pd(OAc)_2_.

Further batch pervaporation studies were undertaken at 65 °C with crosslinked PIM-1 membranes, with initial feed compositions of 87, 77, 60, 50, 40, 30, 20, and 10 vol % toluene, taking up to three samples at 2 h intervals. This gave data over a wide range of feed compositions, from 87 vol % toluene down to 10 vol % toluene. Figure 8a shows values obtained for toluene flux and DMSO flux, and Figure 8b shows values of toluene/DMSO separation factor, over the range of feed compositions. There is a general increase in separation factor as the amount of toluene in the feed decreases, although there is scatter in the data due to difficulty in quantifying the small amount of DMSO in the permeate.

**Figure 9:**
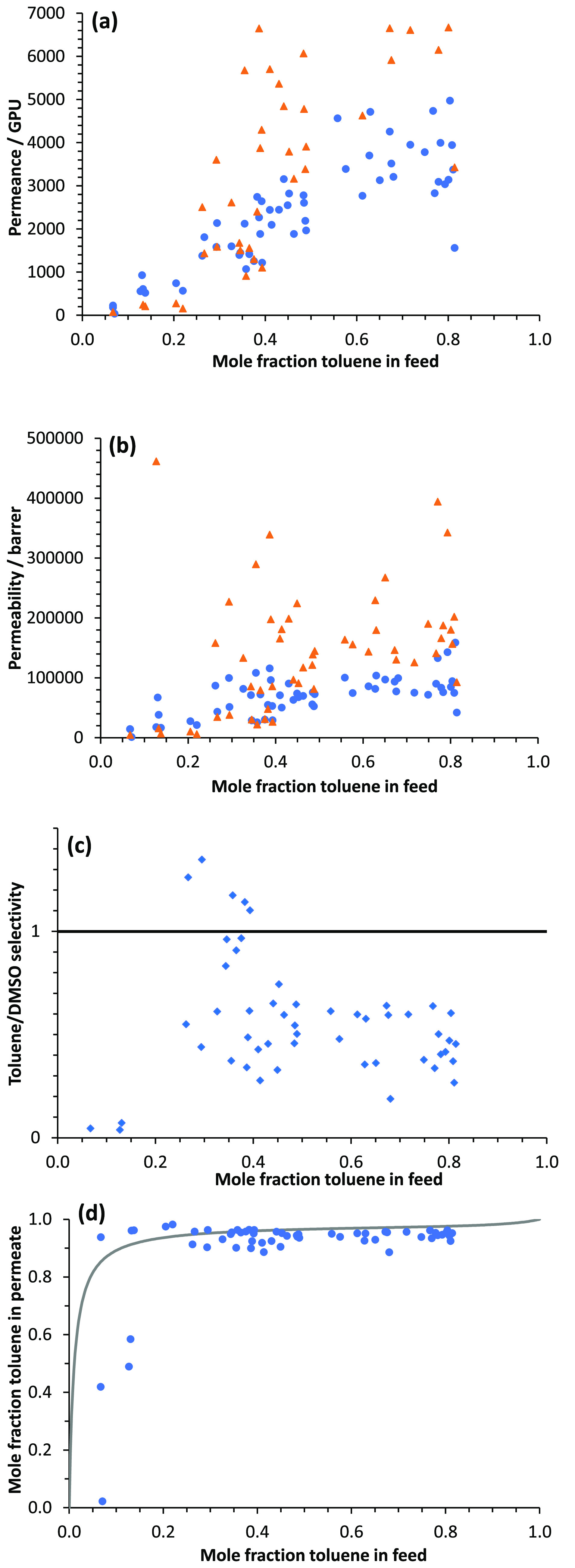
Dependence of (a) toluene permeance (blue circle solid) and DMSO permeance (orange triangle solid), (b) toluene permeability (blue circle solid) and DMSO permeability (orange triangle solid), and (c) toluene/DMSO selectivity on mole fraction of toluene in the feed (average over the time period of permeate collection) for pervaporation at 65 °C using branched PIM-1 membranes crosslinked with Pd(OAc)_2_. (d) McCabe–Thiele plot of mole fraction toluene in permeate against mole fraction toluene in feed, comparing experimental pervaporation data (blue circle solid) with vapor–liquid equilibrium curve for toluene/DMSO at 65 °C (**—**).

Figure 9a shows the effect of converting flux to permeance, taking account of the driving force. Although the DMSO fluxes are very low, the DMSO permeances are similar to or higher than those for toluene, due to the very small driving force for DMSO permeation. Figure 9b shows the effect of converting permeance to permeability, based on the unswollen membrane thickness. The toluene permeability is reasonably constant, in the approximate range 50 000–100 000 barrer, at toluene mole fractions above about 0.3. There is considerable scatter in the DMSO permeability data, which perhaps reflects differing degrees of swelling of the membranes. The difficulty in getting good permeability data when a component is present in a very small amount in the permeate, and the membrane may be swollen to an unknown extent, perhaps helps to explain why such conversions are often not carried out in the pervaporation literature. Figure 9c shows values obtained for toluene/DMSO membrane selectivity over the range of feed compositions. For most of the points, the selectivity is <1, confirming that the membrane is if anything slightly selective for DMSO over toluene and that it is the high driving force for toluene evaporation that leads to high separation factors for toluene over DMSO, as discussed above.

The performance in pervaporation is conveniently compared with vapor–liquid equilibrium in a McCabe–Thiele plot of mole fraction in permeate against mole fraction in feed, shown in Figure 9d. The vapor–liquid equilibrium curve for toluene/DMSO at 65 °C was calculated using ASPEN PLUS software. The strong driving force for toluene evaporation means that there is little scope for improving the separation by pervaporation. However, there may be an advantage of pervaporation over a process such as distillation in terms of energy costs because pervaporation can be conducted at relatively low temperatures.

## 4 Conclusions

Polymerizations carried out at the same set temperature may give different results because of differences in the actual temperature profile over the course of the reaction. For PIM-1 formed by polymerization of TTSBI with TFTPN at a set temperature of 160 °C, instances of monosubstitution leading to branched structures may occur under conditions, such as higher dilution, that lower the temperature in the early stages of reaction. In solution in chloroform, branched versions of PIM-1 prepared in this way show similar hydrodynamic behavior to linear disubstituted PIM-1, with similar Mark–Houwink coefficients. However, they interact strongly with Pd(OAc)_2_, forming a gel.

Membranes of branched PIM-1 and branched PIM-Py cast from chloroform may be crosslinked under ambient conditions by immersion in methanol in the presence of Pd(OAc)_2_. The crosslinked membranes are sufficiently stable in organic solvents to be used for the pervaporative removal at 65 °C of toluene from mixtures with DMSO. Both PIM-1 and PIM-Py membranes give higher toluene/DMSO separation factors than those achieved previously with membranes based on PIM-SBF (with a 77 vol % toluene feed, β = 10 for PIM-Py). However, the membranes themselves are slightly selective for DMSO and it is the high driving force for toluene evaporation that drives the separation.

The ability to crosslink membranes of branched PIMs under ambient conditions opens up many possibilities for the separation of organic/organic mixtures.

## Data Availability

Data supporting this study are available within the article and Supporting Information.

## Supporting Information Available

Materials used, purification, and characterization of the polymers; summary of polymer synthesis and historical branched PIM-1 polymers; ^1^H NMR spectra of the PIM polymers; examples of Lorentz peak fitting of aromatic proton region of polymer spectra; tables and information detailing the determination of branching present (%) in each polymer sample; IR spectra of PIM-1 films; elemental analyses of crosslinked PIM membranes; and tables of pervaporation results from crosslinked PIM-1 and PIM-Py membranes (PDF)

## Abbreviations

PIM: polymer of intrinsic microporosity
DMSO: dimethyl sulfoxide
PEI: polyethyleneimine
Pd(OAc)_2_: palladium(II) acetate
BTX: benzene, toluene and xylenes
TTSBI: 5,5′6,6′-tetrahydroxy-3,3,3′,3′-tetramethyl-1,1′-spirobisindane
TFTPN: tetrafluoroterephthalonitrile
TFPCN: 2,3,5,6-tetrafluoro-4-pyridinecarbonitrile
PIM-1: polymer formed from TTSBI and TFTPN
PIM-Py: polymer formed from TTSBI and TFPCN
PIM-SBF: polymer formed from 2,2′,3,3′-tetrahydroxy-9,9′-spirobifluorene and TFTPN
SEC: size exclusion chromatography
GC: gas chromatography
^1^H NMR: proton nuclear magnetic resonance spectroscopy
FTIR: Fourier transform infrared spectroscopy
