# Supplementary material for: Crosslinking of Branched PIM-1 and PIM-Py Membranes for Recovery of Toluene from Dimethyl Sulfoxide by Pervaporation
Source: ACS Appl Polym Mater. 2023 Jan 17;5(2):1145–58. doi: 10.1021/acsapm.2c01600 (PMC9926464; doi:10.1021/acsapm.2c01600)
Supplement: Supplementary file 1 — ap2c01600_si_001.pdf [file ap2c01600_si_001.pdf]

Supporting Information

Crosslinking of branched PIM-1 and PIM-Py membranes for recovery of toluene from dimethyl sulfoxide by pervaporation

Sulaiman Aloraini[1,2], Michael Mathias[1], Jessica Crone[1], Kurtis Bryce[1], Ming Yu[1,3], Richard A. Kirk[1], Mohd Zamidi Ahmad[1], Edidiong D. Asuquo[1], Sandra Rico-Martínez[4], Alexey V. Volkov[5,†], Andrew B. Foster[1] and Peter M. Budd[1]**

AUTHOR INFORMATION

[1]Department of Chemistry, University of Manchester, Oxford Road, Manchester M13 9PL, United Kingdom.

[2]Department of Chemistry, College of Science and Arts, Qassim University, Ar Rass, Saudi Arabia.

[3]Department of Chemical Engineering, The University of Melbourne, Melbourne, VIC. 3010, Australia.

[4]IU CINQUIMA, Universidad de Valladolid, Paseo Belén 5, E-47011 Valladolid, Spain.

[5]A.V. Topchiev Institute of Petrochemical Synthesis, 29 Leninsky Av., Moscow 119991, Russian Federation.

†Present address: Chemistry Program, Physical Science Program, Environmental Science and Engineering Program, Biological and Environmental Science and Engineering Division (BESE), Advanced Membranes and Porous Materials (AMPM) Center, King Abdullah University of Science and Technology (KAUST), 23955-6900 Thuwal, Saudi Arabia.

Corresponding Authors*

Andrew B. Foster – Department of Chemistry, University of Manchester, Oxford Road, Manchester M13 9PL, United Kingdom; Email: andrew.foster@manchester.ac.uk.

Peter M. Budd – Department of Chemistry, University of Manchester, Oxford Road, Manchester M13 9PL, United Kingdom; Email: peter.budd@manchester.ac.uk.

KEYWORDS Polymer of intrinsic microporosity, crosslinking, pervaporation, organic-organic separation, branched PIMs.

TABLE OF CONTENTS

**S1.** Materials.	S4
**Table S1.** Nitrogen purged, PIM polymerizations.	S5
**S2.** Purification of the Polymers.	S6
**S3.** Polymer Characterization.	S6
**S4.** Historical branched PIM-1 samples.	S8
**Table S2.** Historical branched PIM-1 characterization data.	S9
**Figure S1.** ^{1}H NMR spectrum of very high molar mass PIM-1 polymer (**1**, M_w = 1,075,000, *Đ* = 4.5) synthesized with extra solvent added during the polymerization (0.05 mol scale).	S9
**Figure S2.** ^{1}H NMR spectrum of PIM-1 polymer (**2**, M_w = 127,000, *Đ* = 2.0) synthesized in very large-scale polymerization (0.5 mol scale).	S10
**Figure S3.** ^{1}H NMR spectrum of PIM-1 polymer (**3**, M_w = 116,300, *Đ* = 2.0) synthesized in DMAc/toluene (20 vol % excess at start) with extra solvent added during the polymerization, at average temperature of 141 °C (0.05 mol scale).	S10
**Figure S4.** ^{1}H NMR spectrum of PIM-1 polymer (**4**, M_w = 142,600, *Đ* = 2.6) synthesized in DMAc/toluene (20 vol % excess at start) with extra solvent added during the polymerization, at average temperature of 127 °C (0.05 mol scale).	S11

Figure S5. ^{1}H NMR spectrum of PIM-1 polymer (**5**, M_w = 119,200, $Đ$ = 2.2) synthesized in DMAc/DCB (20 vol % excess at start) with extra solvent added during the polymerization (0.05 mol scale). S11

Figure S6. ^{1}H NMR spectrum of PIM-1 polymer (**6**, M_w = 187,600, $Đ$ = 2.1) synthesized in DMAc/toluene (20 vol % excess at start) with extra solvent added during the polymerization (0.05 mol scale). S12

Figure S7. ^{1}H NMR spectrum of PIM-Py polymer (**8**, M_w = 223,200, $Đ$ = 6.7) synthesized in DMF at 65 °C in 3 days (0.15 mol scale). S12

Figure S8. Lorentz peak fitting of aromatic proton region of ^{1}H NMR spectrum of PIM-1 polymer (**5**, M_w = 119,200, $Đ$ = 2.2). S13

Figure S9. Lorentz peak fitting of aromatic proton region of ^{1}H NMR spectrum of PIM-1 polymer (**6**, M_w = 187,600, $Đ$ = 2.1). S13

Figure S10. Lorentz peak fitting of aromatic proton region of ^{1}H NMR spectrum of PIM-1 polymer (**7**, M_w = 107,500, $Đ$ = 1.9). S14

Figure S11. Lorentz peak fitting of aromatic proton region of ^{1}H NMR spectrum of PIM-Py polymer (**8**, M_w = 223,200, $Đ$ = 6.7). S14

Table S3. Estimation of amount of branching present in PIM-1 polymer samples (**1**–**7**) from Lorentz peak fitting of the aromatic proton regions in their respective ^{1}H NMR spectra. S15

Table S4. Estimation of amount of branching present in PIM-Py polymer sample (**8**) from Lorentz peak fitting of the aromatic proton region of ^{1}H NMR spectrum. S16

Figure S12. (a) Branched PIM-1 sample **6** in chloroform entirely gelled by addition of palladium (II) acetate. **(b)** Aromatic proton NMR region of branched PIM-1 sample **6** (bottom) and remnant of PIM-1 in solution after crosslinking with palladium (II) acetate (top). **(c)** FT-IR spectrum of solid state $Pd(OAc)_2$ crosslinked PIM-1 **6** film, crosslinked in the solid state with $Pd(OAc)_2$, after chloroform washings. S16

Figure S13. FT-IR spectrum of PIM-1 film (**5**). S17

Figure S14. FT-IR spectrum of solution state $Pd(OAc)_2$ treated (50 mol %) PIM-1 film (**5**). S17

Table S5. Conditions employed and elemental analyses of PIM polymer membranes before and after treatment with palladium acetate to crosslink the films (xPIM-1 and xPIM-Py) for pervaporation studies. S18

Table S6. Pervaporation results for PIM-1 membranes crosslinked with $Pd(OAc)_2$. S19

Table S7. Pervaporation results for PIM-Py membranes crosslinked with $Pd(OAc)_2$. S22

S1. Materials.

The monomers, 5,5′,6,6′-tetrahydroxy-3,3,3′,3′-tetramethyl-1,1′- spirobisindane (TTSBI), tetrafluoroterephthalonitrile (TFTPN, 100 %) and 2,3,5,6-tetrafluoro-4-pyridinecarbonitrile (TFPCN, 99.3 %) were purchased from Alfa Aesar, Fluorochem and Sigma-Aldrich, respectively. Both TFTPN and TFPCN were used as received at certified levels of purity, after drying in vacuum overnight at room temperature. TTSBI required further purification before use. TTSBI was stirred in ethyl acetate at a concentration of 1 g mL^{-1} in a round bottom flask under N_2 and heated to reflux. Ethyl acetate was added in small aliquots until the TTSBI was fully solubilized. While the solution was still under reflux, an equal amount of hexane was added to the solution while hot and further stirred for 10 min. The solution was cooled to room temperature and then cooled to −5 °C, using a water/dry ice mixture for 4 h. The resulting white precipitate was collected via filtration and washed with hexane to remove any traces of ethyl acetate. After drying under nitrogen for 1 h, the white solid was dried under vacuum at room temperature overnight to avoid oxidation, to afford the desired product as a fine white powder.

Anhydrous potassium carbonate (K_2CO_3, 99.5%) was purchased from Fisher Scientific Ltd, with the base ground into a fine powder and dried in a vacuum oven at 110 °C overnight before use. Anhydrous dimethylformamide (DMF), anhydrous dimethylacetamide, (DMAc), toluene, dimethyl sulfoxide (DMSO), methanol, chloroform, tetrahydrofuran, 1,4-dioxane and palladium (II) acetate were purchased from Sigma-Aldrich and used as received.

Table S1. Nitrogen-purged, PIM polymerizations.

Polymerization[a]	Solvent mixture (2:1 by volume)	Reaction scale (mol)	Polymer concentration[b] (mol dm^{-3})	Set [average] temperature (°C)	Reaction time (min)	Yield (%)
PIM-1 **1**	DMAc/toluene	0.05	0.33 / [c]	160 [[c]]	60	68
PIM-1 **2**	DMAc/toluene	0.5	0.33	160 [[c]]	38	94
PIM-1 **3**	DMAc/toluene	0.05	0.27 / 0.21	160 [141]	30	97
PIM-1 **4**	DMAc/toluene	0.05	0.27 / 0.21	160 [127]	40	97
PIM-1 **5**	DMAc/DCB	0.05	0.27 / 0.21	140 [[c]]	120	97
PIM-1 **6**	DMAc/toluene	0.05	0.27 / 0.21	160 [>130]	60	98
PIM-1 **7**	DMAc/toluene	0.05	0.27 / 0.21	160 [129]	36	91
PIM-Py **8**	DMF	0.15	0.25	65 [65]	3 days	97

[a] PIM-1 polymerizations were generally carried out in DMAc/toluene solvent mixtures (2:1 by volume) in the presence of three-fold molar excess of potassium carbonate. Exception was reaction **5** which was carried out in DMAc/dichlorobenzene (DCB) solvent mixture (2:1 by volume). PIM-Py polymerization was carried out in dimethylformamide (DMF); [b] Potential polymer concentration at start/end of the reaction after additions of extra solvent mixture; [c] information not available.

S2. Purification of the Polymers.

Each recovered filtered polymer was re-dissolved in $CHCl_3$ (concentration of 5 g in 120 mL) and re-precipitated by pouring slowly into excess methanol. The polymer was collected via filtration and refluxed in de-ionized water for 16 h. After refluxing in water, the mixture was filtered by vacuum filtration and then immersed in a minimal amount of 1,4-dioxane for 15 min (volume used was just enough to cover the mass of polymer in the beaker) to remove low molecular weight oligomers. The polymer was vacuum filtered before washing with excess of acetone to remove traces of 1,4-dioxane. The filtered polymer was then soaked in methanol for 12 h to remove all traces of dioxane and acetone. Finally, the polymer was filtered dry using a sintered funnel under vacuum, before drying in a vacuum oven at 120 °C for 72 h to remove all trace solvents.

S3. Polymer Characterization.

Size exclusion chromatography (SEC) analysis: Average molar masses of the polymers were measured by triple detector size exclusion chromatography (SEC). Analysis was performed in chloroform from 1 mg mL^{-1} polymer solutions (injection volume 100 µL) at a flow rate of 1 mL min^{-1} using a Viscotek VE2001 SEC solvent / sample module with two PL Mixed B columns maintained at 35 °C and a Viscotek TDA 302 triple detector array (refractive index, light scattering, viscosity detectors). The data were analysed in OmniSec software.

Nuclear Magnetic Resonance (NMR) analysis: ^1H NMR spectra of the polymers were recorded using a Bruker Avance II 500 MHz instrument. 50 mg mL^{-1} polymer solutions in $CDCl_3$ were prepared for the NMR analysis. Signal peaks for the solvent were used as references. An example of a ^1H NMR spectrum of a strongly branched PIM-1 polymer sample

(**7**) is presented later in **Figure 3**. The spectra obtained for the other PIM samples are provided in **Figures S1−7**. Lorentz peak fitting of the aromatic proton region (δ = 6.0−7.2 ppm) of each proton NMR spectrum was used to determine the respective integral areas associated with resonances attributed to disubstituted PIM-1 residue and branch point stuctures (aromatic protons labelled **a**, **b**, **c** and **d** in **Figure 1**). Examples of the peak fittings obtained for the aromatic proton regions of ^{1}H NMR spectra of polymers, **5**, **6**, **7** and **8** are presented in **Figures S8−11**. This allowed an estimation of the percentage of branch points present in each PIM polymer sample as a proportion of all residues present (calculations further outlined in **Tables S3−S4** and results presented in **Table 1**).

Determination of network content. A polymer solution in chloroform (1 mg mL^{-1}) was accurately prepared from 10-15 mg of a polymer sample. The entire solution was passed through a 0.45 μm PTFE syringe filter and the exact weight of solution collected into a 30 mL sample bottle was recorded. The solvent in the bottle was allowed to slowly evaporate over several days. Once the solvent had visually completely evaporated, the bottle was placed in an oven at 100 °C to complete the drying process. The weight of polymer remaining in the bottle was measured and compared against the mass of filtered solvent (volume of chloroform) initially collected to determine the filtered polymer concentration. The network content by filtration was determined as the difference between the initial and filtered concentrations of the polymer as a percentage.

Elemental analysis: A Flash 2000 Organic Elemental Analyser (Thermo Scientific, The Netherlands) was employed to obtain elemental analysis (C, H, N) data. 1-2 mg of polymer film was used for each experiment. Fluorine (F) analysis of some polymer samples was carried out by Exeter Analytical UK Ltd. It was carried out by combustion of an accurately weighed sample in

a silica oxygen flask over an accurately measured volume of water and total ionic strength adjustment buffer (TISAB) solution. A fluoride ion selective electrode was used to measure the potential of a range of fluoride solutions. The potential of the combusted samples was measured under the same conditions. Pd analysis was done by weighing between 1-20 mg of the polymer film and acid digesting it in a mixture of nitric and perchloric acid, up to 200°C max. After acid digestion, hydrochloric acid was added to dissolve any residue and then this solution was diluted with deionised water and made up to a known volume in a volumetric flask. A Thermo Scientific iCAP 6300 Duo ICP-OES instrument was used to measure the concentration (in ppm) of Pd in the aqueous solution, and then by back calculation the amount of Pd (%) present in the original film sample was determined.

Fourier-Transform Infrared (FTIR) Spectroscopy: Analysis was performed on a Perkin Elmer FTIR at a wavelength range of 600 – 4000 cm^{-1} and a resolution of 4 cm^{-1}.

S4. Historical branched PIM-1 samples.

Historical polymerizations **1** and **2** involved minor variations on conditions previously utilized for PIM-1 synthesis and their ^{1}H NMR spectra indicate a significant amount of branched structures, 13.5 % and 6.5 %, respectively (**Figures S1–2**). For polymerization **1**, an unspecified amount of additional solvent was added during the polymerization to lower the viscosity of the reaction mixture, which is also likely to have lowered the temperature profile during the polymerization. Polymerization **2** was carried out on a larger scale (0.5 mol rather than 0.05 mol), which is likely to have led to a slower rate of heating than when carried out at the smaller scale, so that the early stages of polymerization occurred at lower temperatures.

Table S2. Historical branched PIM-1 characterization data.

Polymer	^{1}H NMR Branching (%)[a]	Multi-detector SEC M_w (kg mol^{-1})	$Đ$
PIM-1 **1**	13.5	1075	4.5
PIM-1 **2**	6.5	127.0	2.0

[a] Defect peaks attributed to branch points compared as percentage of major peaks attributed to disubstituted PIM residue structures as outlined in **Table S3**.

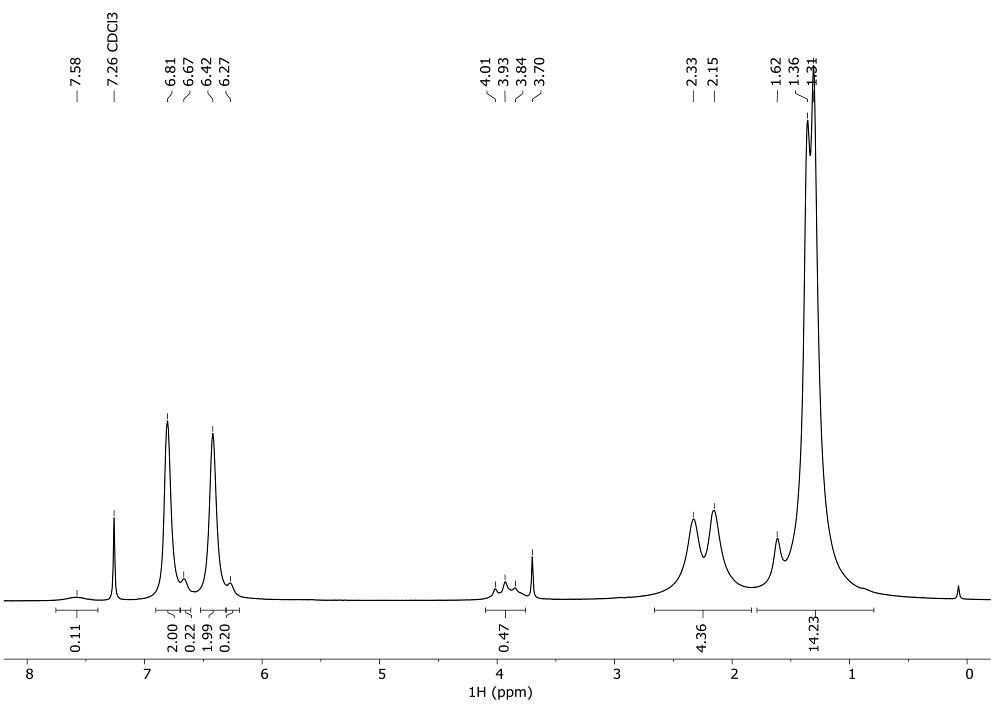

Figure S1. ^{1}H NMR spectrum of very high molar mass PIM-1 polymer (**1**, M_w = 1,075,000, $Đ$ = 4.5) synthesized with extra solvent added during the polymerization (0.05 mol scale).

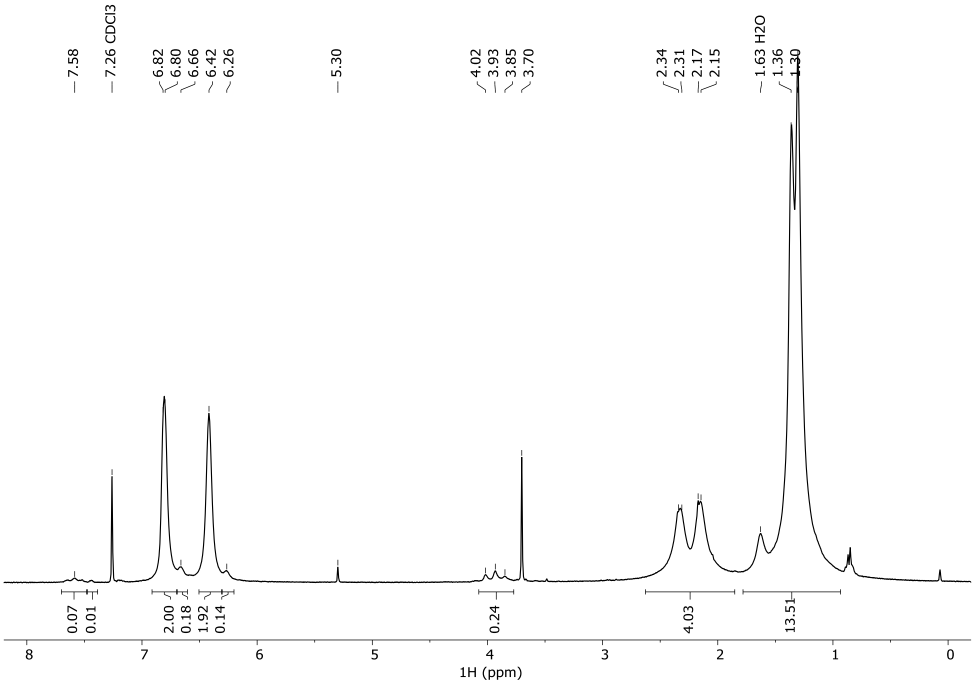

Figure S2. ^{1}H NMR spectrum of PIM-1 polymer (**2**, M_w = 127,000, *Đ* = 2.0) synthesized in very large-scale polymerization (0.5 mol scale).

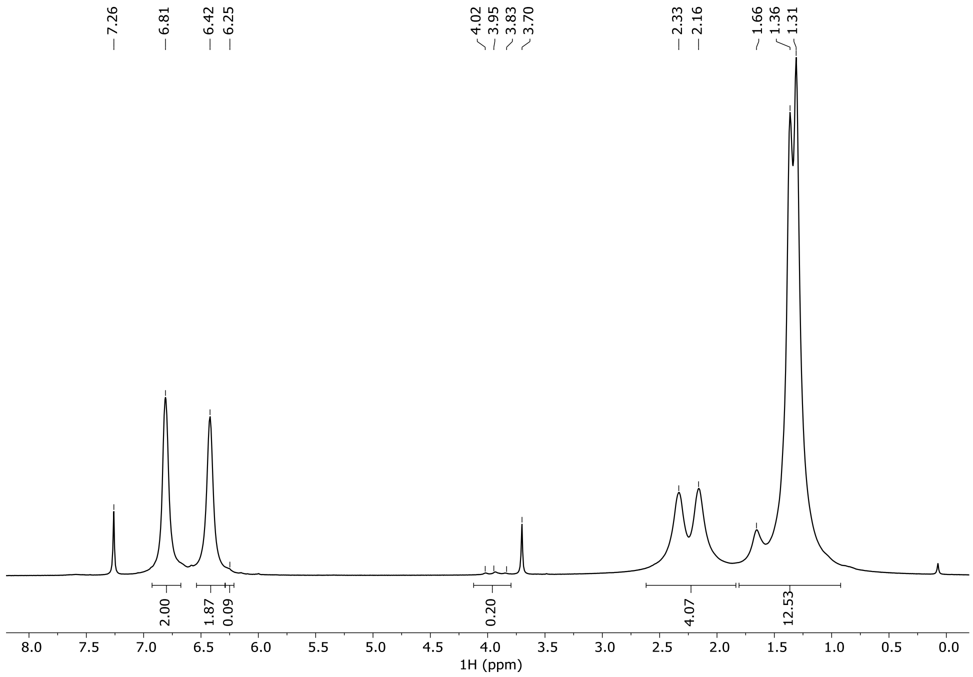

Figure S3. ^{1}H NMR spectrum of PIM-1 polymer (**3**, M_w = 116,300, *Đ* = 2.0) synthesized in DMAc/toluene (20 vol % excess at start) with extra solvent added during the polymerization, at average temperature of 141 °C (0.05 mol scale).

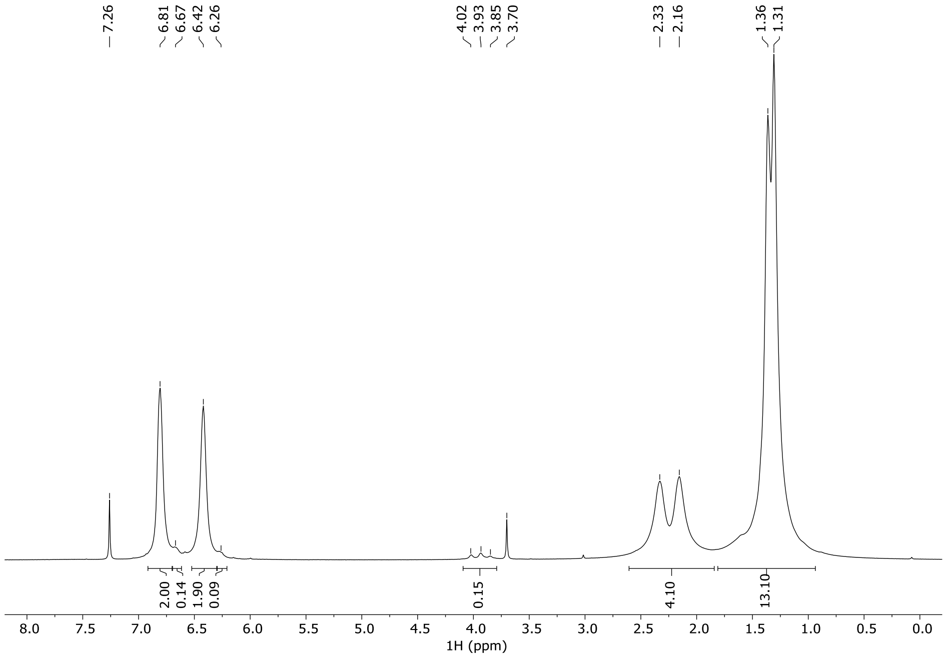

Figure S4. ^{1}H NMR spectrum of PIM-1 polymer (**4**, M_w = 142,600, $Đ$ = 2.6) synthesized in DMAc/toluene (20 vol % excess at start) with extra solvent added during the polymerization, at average temperature of 127 °C (0.05 mol scale).

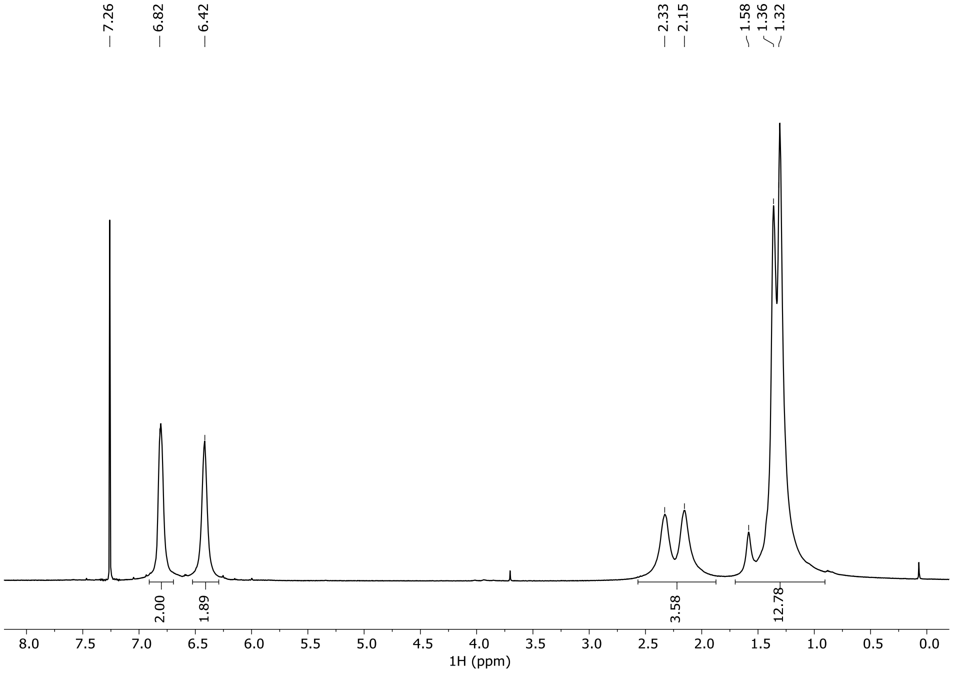

Figure S5. ^{1}H NMR spectrum of PIM-1 polymer (**5**, M_w = 119,200, $Đ$ = 2.2) synthesized in DMAc/DCB (20 vol % excess at start) with extra solvent added during the polymerization (0.05 mol scale).

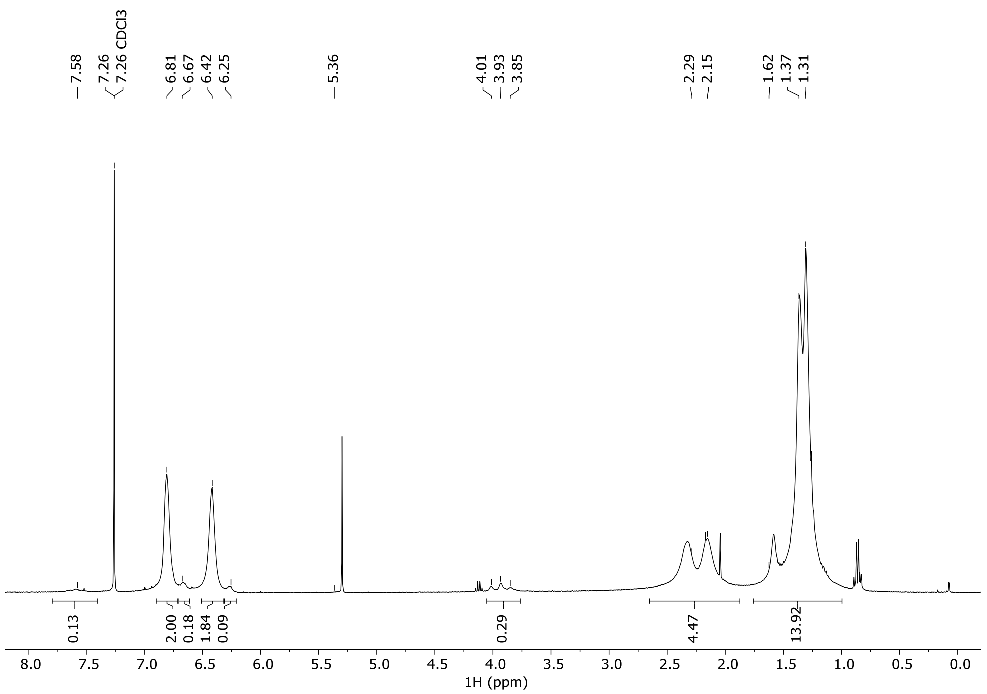

Figure S6. ^{1}H NMR spectrum of PIM-1 polymer (**6**, M_w = 187,600, $Đ$ = 2.1) synthesized in DMAc/toluene (20 vol % excess at start) with extra solvent added during the polymerization (0.05 mol scale).

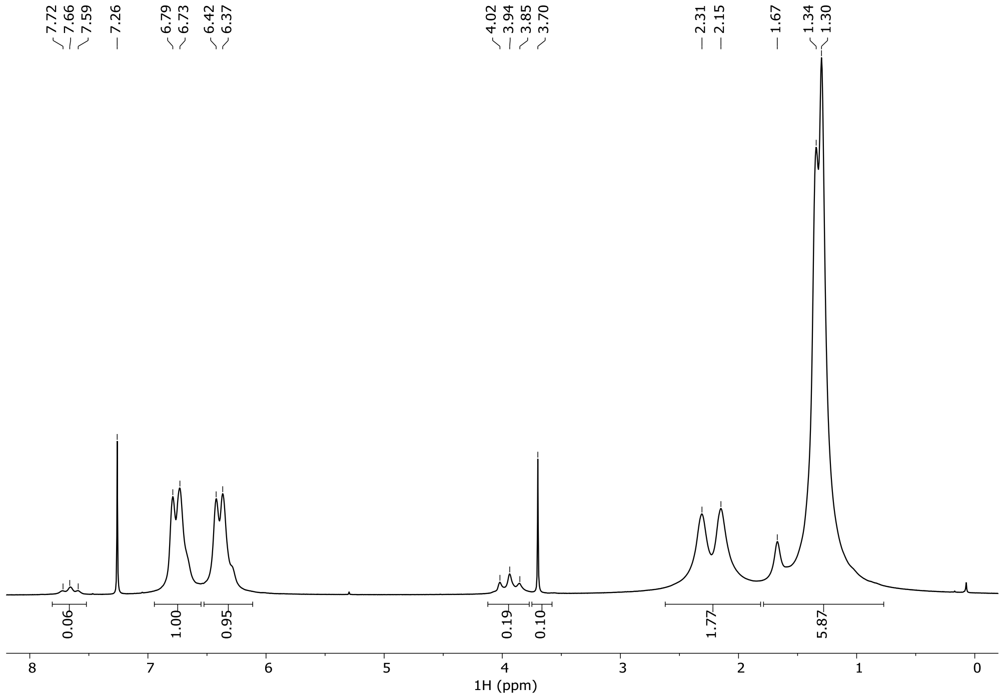

Figure S7. ^{1}H NMR spectrum of PIM-Py polymer (**8**, M_w = 223,200, $Đ$ = 6.7) synthesized in DMF at 65 °C in 3 days (0.15 mol scale).

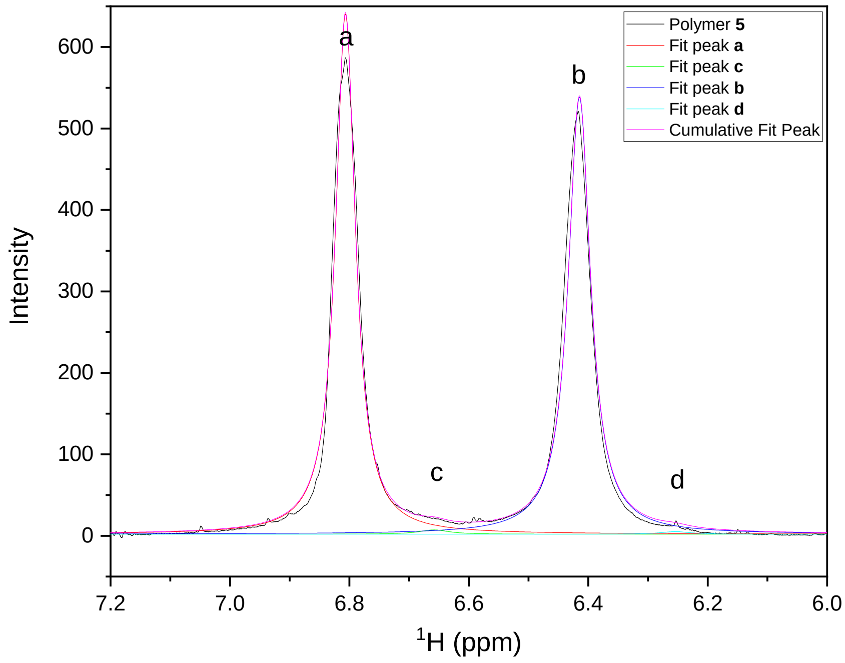

Figure S8. Lorentz peak fitting of aromatic proton region of ^{1}H NMR spectrum of PIM-1 polymer (**5**, M_w = 119,200, $Đ$ = 2.2).

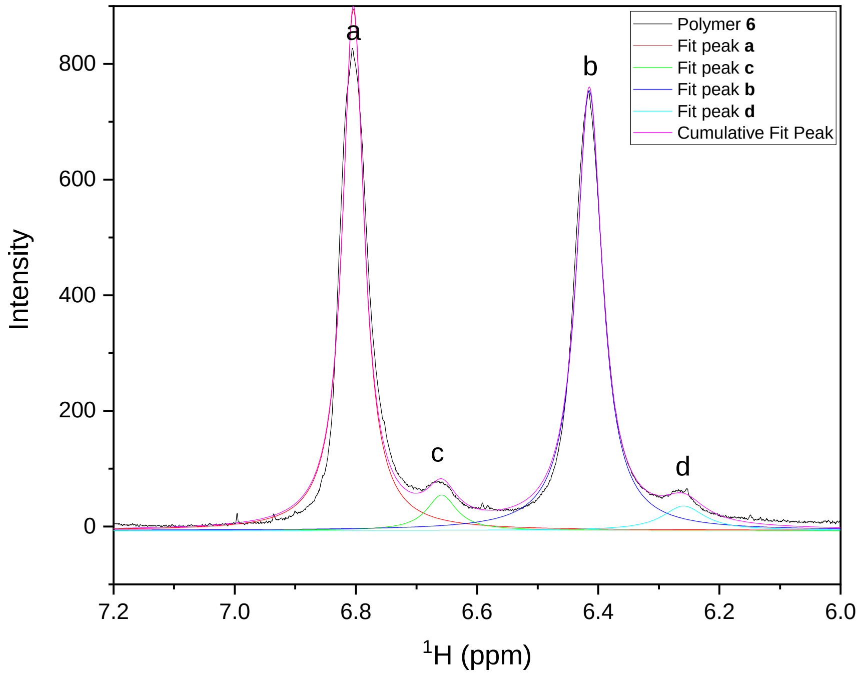

Figure S9. Lorentz peak fitting of aromatic proton region of ^{1}H NMR spectrum of PIM-1 polymer (**6**, M_w = 187,600, $Đ$ = 2.1).

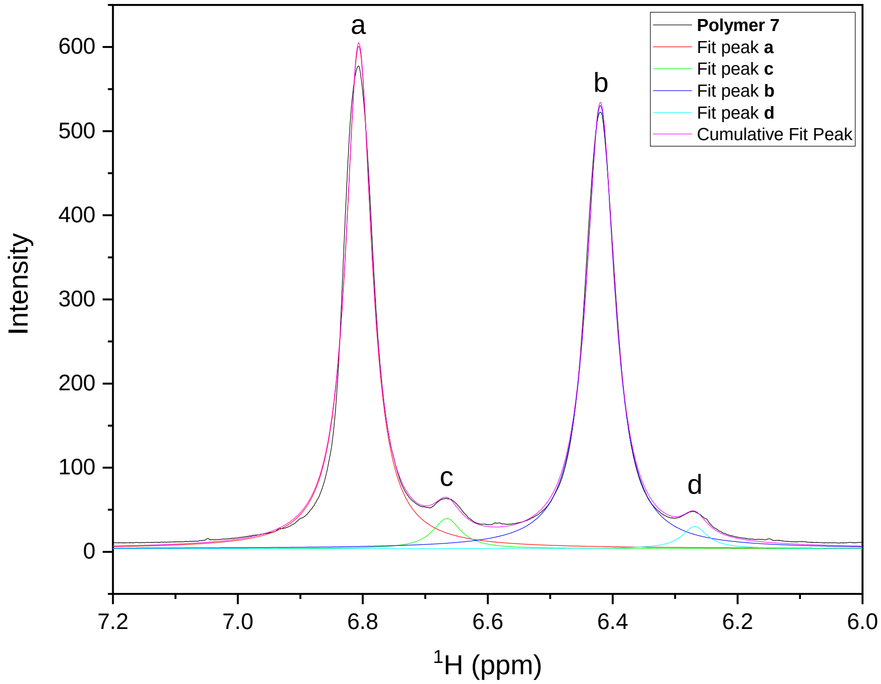

Figure S10. Lorentz peak fitting of aromatic proton region of ^{1}H NMR spectrum of PIM-1 polymer (**7**, M_w = 107,500, $Đ$ = 1.9).

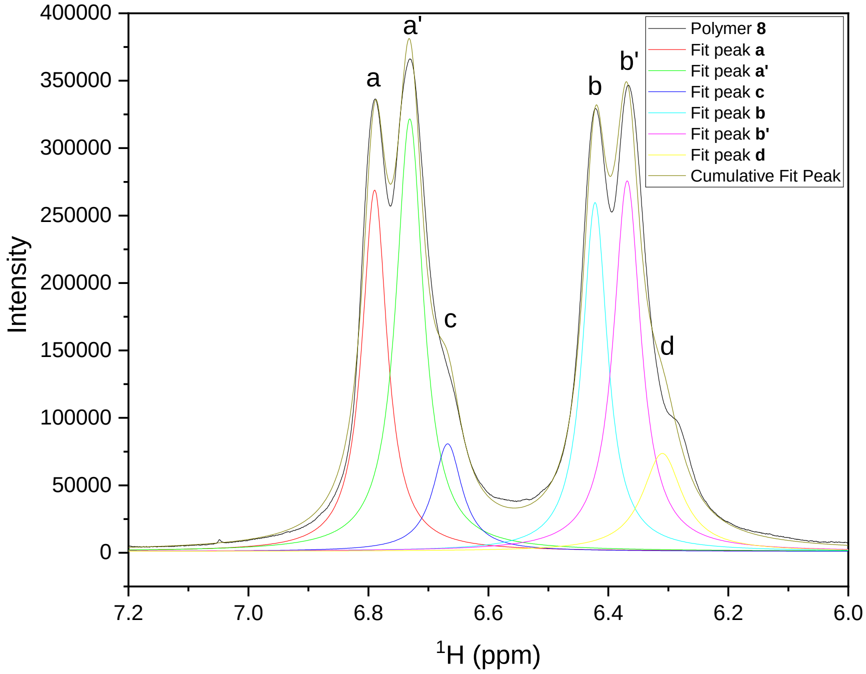

Figure S11. Lorentz peak fitting of aromatic proton region of ^{1}H NMR spectrum of PIM-Py polymer (**8**, M_w = 223,200, $Đ$ = 6.7).

Table S3. Estimation of amount of branching present in PIM-1 polymer samples (**1–7**) from Lorentz peak fitting of the aromatic proton regions in their respective ^{1}H NMR spectra.

Polymer	Integral area (*I*) of respective aromatic peak contributions				Total integral areas attributed to PIM-1 residues[a] and branch points[b]		Branching[c] (%)	PIM-1 residues per branch point[d]
	I_a	I_b	I_c	I_d	I_t (PIM-1)	I_t (branch)		
**1**	61.916	62.405	4.796	4.203	115.32	18.00	13.5	6.4
**2**	68.308	68.308	2.584	2.025	132.01	9.218	6.5	14.3
**3**	47.827	45.370	1.219	0.544	91.43	3.526	3.7	26.0
**4**	50.897	48.486	1.913	1.434	96.04	6.694	6.5	14.3
**5**	40.116	37.907	0.500[e]	0.300[e]	77.22	1.600	~2.0[e]	48[e]
**6**	60.898	61.822	5.770	5.634	111.32	22.81	17.0	4.9
**7**	47.415	45.473	3.100	2.031	87.757	10.262	10.5	8.6

[a] Total integral area attributed to four aromatic protons associated with disubstituted PIM-1 residue structures, I_t (PIM-1) = sum of integral areas of aromatic peaks, **a** + **b**, minus the contribution of other two unresolved aromatic protons (equivalent to integrals of **c** + **d**) associated with the branching structure.

[b] Total integral area attributed to four aromatic protons associated with branch points, I_t (branch) = sum of integral areas of peaks, **c** + **d**, multiplied by two to include the contributions for the other two unresolved aromatic protons.

[c] Branch points as percentage of all residues evident in polymer, branched (%) = I_t (branch) / [I_t (PIM-1) + I_t (branch)] × 100.

[d] PIM-1 residues per branch point = [100 – Branched (%)] / Branched (%).

[e] Aromatic peaks, **c** and **d**, associated with branch point, are very small and not clearly resolvable from the main aromatic band peaks, **a** and **b**.

Table S4. Estimation of amount of branching present in PIM-Py polymer sample (**8**) from Lorentz peak fitting of the aromatic proton region of ^{1}H NMR spectrum.

Polymer	Integral area (I) of respective aromatic peak contributions			Total integral areas attributed to PIM-Py residues[a] and branch points[b]		Branching[c] (%)	PIM-Py residues per branch point[d]
	I_a	$I_{a'}$	I_c	I_t (PIM-Py)	I_t (branch)		
**8**	21.047	27.713	7.272	41.488	14.544	26.0	2.8

[a] Total integral area attributed to two aromatic protons associated with disubstituted PIM-Py residue structures, I_t (PIM-Py) = sum of integral areas of aromatic peaks, **a** + **a′**, minus the contribution of unresolved aromatic proton (equivalent to integral of **c**) associated with the branching structure.

[b] Total integral area attributed to two aromatic protons associated with branch points, I_t (branch) = integral area of peak, **c**, multiplied by two to include the contribution for the other unresolved aromatic proton.

[c] Branch points as percentage of all residues evident in polymer, branched (%) = I_t (branch) / [I_t (PIM-Py) + I_t (branch)] × 100.

[d] PIM-Py residues per branch point = [100 – Branched (%)] / Branched (%).

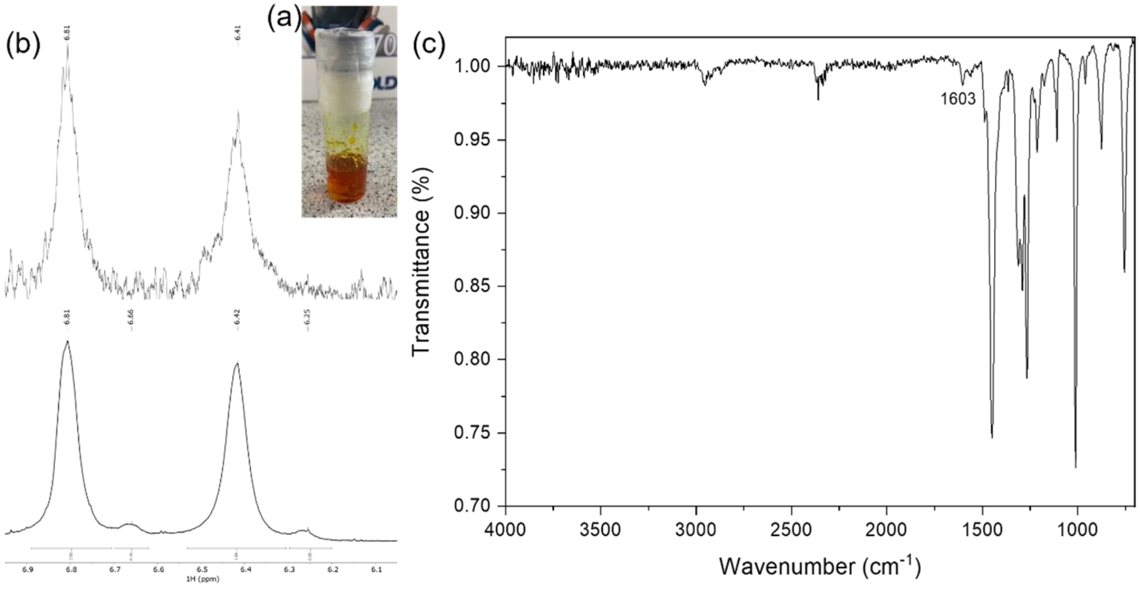

Figure S12. (a) Branched PIM-1 sample **6** in chloroform entirely gelled by addition of palladium (II) acetate. **(b)** Aromatic proton NMR region of branched PIM-1 sample **6** (bottom) and remnant of PIM-1 in solution after crosslinking with palladium (II) acetate (top). **(c)** FT-IR spectrum of solid state $Pd(OAc)_2$ crosslinked PIM-1 **6** film, crosslinked in the solid state with $Pd(OAc)_2$, after chloroform washings.

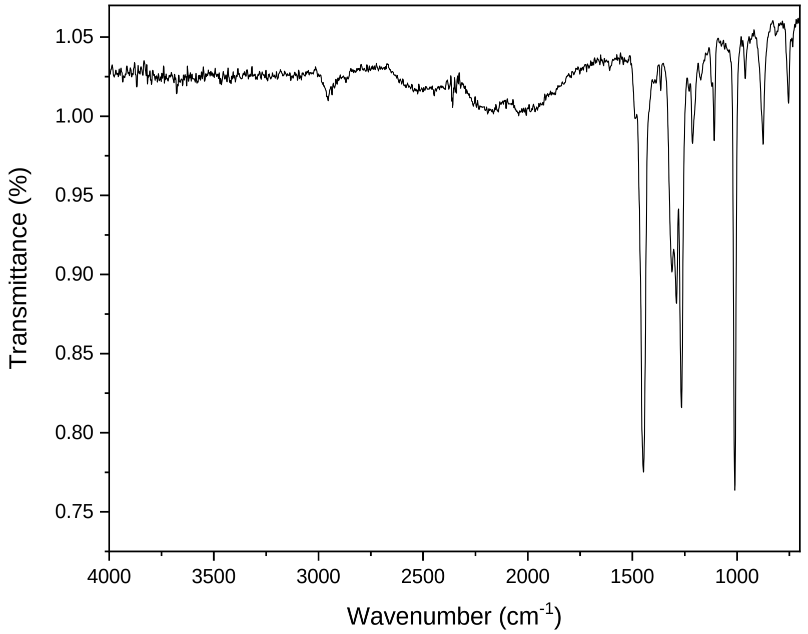

Figure S13. FT-IR spectrum of PIM-1 film (**5**).

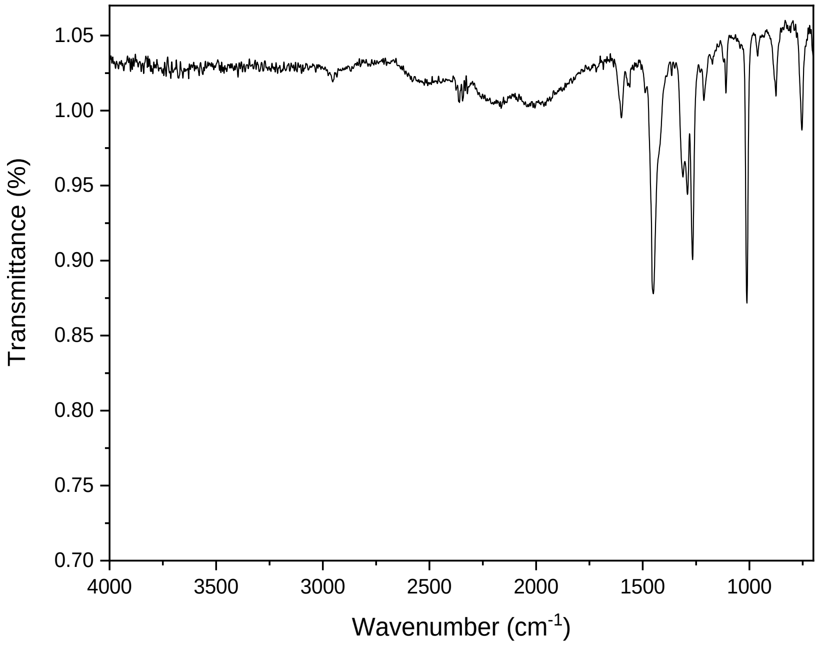

Figure S14. FT-IR spectrum of solution state $Pd(OAc)_2$ treated (50 mol %) PIM-1 film (**5**).

Table S5. Conditions employed and elemental analyses of PIM polymer membranes before and after treatment with palladium acetate to crosslink the films (xPIM-1 and xPIM-Py) for pervaporation studies.

Polymer form	Treatment	Film thickness Start [end] (μm)	C (%)	N (%)	H (%)	Pd (%)	$Pd(OAc)_2$ (mol %)
PIM-1 **6**	-	-	73.07	5.83	4.42	0	
xPIM-1 **6**	100 mol % $Pd(OAc)_2$, 4 h	40 [20]	-	-	-	2.20	14.2
xPIM-1 **6**	25 mol % $Pd(OAc)_2$, 4 h	60 [42]	71.51	5.73	4.23	1.71	11.0
PIM-1 **7**	-	-	74.01	5.95	4.30	0	
xPIM-1 **7**	25 mol % $Pd(OAc)_2$, 4 h	59 [32]	71.96	5.75	4.39	1.31	8.4
PIM-Py **8**	-	-	73.01	6.29	4.62	0	
xPIM-Py **8**	25 mol % $Pd(OAc)_2$, 4 h	43 [31]	70.15	6.07	4.58	1.84	11.5
xPIM-Py **8**	25 mol % $Pd(OAc)_2$, 4 h	38 [21]	70.90	6.25	4.58	2.16	13.5
xPIM-Py **8**	25 mol % $Pd(OAc)_2$, 4 h	77 [38]	65.84	5.64	4.22	1.40	8.8

Table S6. Pervaporation results for PIM-1 membranes crosslinked with $Pd(OAc)_2$.

Membrane thickness (μm)	Feed initial toluene (vol%)	Time (h)	Feed toluene mole fraction	Permeate toluene mole fraction	Total flux (kg m^{-2} h^{-1})	Toluene flux (kg m^{-2} h^{-1})	DMSO flux (kg m^{-2} h^{-1})	Toluene/ DMSO separation factor	Toluene permeance (GPU)	DMSO permeance (GPU)	Toluene/ DMSO selectivity
19	87	2	0.808	0.943	8.87	8.44	0.43	3.92	3947	10622	0.37
19	87	4	0.783	0.945	8.89	8.47	0.42	4.73	3998	9869	0.41
19	87	6	0.748	0.939	8.35	7.91	0.44	5.13	3781	10003	0.38
27	87	2	0.814	0.952	3.48	3.34	0.14	4.54	1562	3430	0.46
27	87	4	0.801	0.953	6.98	6.70	0.28	5.01	3143	6673	0.47
27	87	6	0.779	0.953	6.80	6.53	0.27	5.81	3093	6150	0.50
19	87	2	0.804	0.962	10.95	10.59	0.36	6.16	4976	8237	0.60
19	87	4	0.767	0.962	10.30	9.96	0.34	7.63	4739	7417	0.64
19	87	6	0.717	0.957	8.47	8.16	0.31	8.72	3952	6612	0.60
47	87	2	0.811	0.925	7.73	7.24	0.50	2.87	3378	12656	0.27
47	87	4	0.793	0.947	6.76	6.46	0.31	4.64	3038	7288	0.42
47	87	6	0.770	0.934	6.33	5.97	0.36	4.24	2831	8386	0.34
31	77	2	0.680	0.886	7.28	6.57	0.72	3.65	3209	17009	0.19
31	77	4	0.650	0.929	6.73	6.32	0.41	7.04	3133	8630	0.36
31	77	6	0.612	0.952	5.73	5.49	0.24	12.46	2771	4631	0.60

22	77	2	0.676	0.955	7.45	7.16	0.29	10.10	3518	5916	0.59
22	77	4	0.630	0.951	9.83	9.42	0.41	11.40	4717	8176	0.58
22	77	6	0.558	0.950	9.21	8.81	0.39	15.06	4566	7437	0.61
22	77	2	0.672	0.957	8.97	8.65	0.33	10.90	4259	6652	0.64
22	77	4	0.628	0.926	7.90	7.40	0.50	7.43	3705	10426	0.36
22	77	6	0.576	0.939	6.98	6.62	0.36	11.41	3393	7080	0.48
29	60	2	0.485	0.940	5.07	4.81	0.26	16.56	2608	4784	0.55
29	60	4	0.449	0.905	4.99	4.58	0.41	11.65	2550	7741	0.33
29	60	6	0.410	0.919	4.55	4.24	0.32	16.27	2443	5705	0.43
24	60	2	0.487	0.948	4.24	4.05	0.19	19.23	2191	3388	0.65
24	60	4	0.452	0.952	5.29	5.07	0.22	24.05	2824	3793	0.74
24	60	6	0.414	0.887	4.05	3.65	0.40	11.08	2100	7540	0.28
37	60	2	0.489	0.936	3.85	3.64	0.21	15.20	1968	3910	0.50
37	60	4	0.463	0.943	3.60	3.42	0.18	19.07	1886	3166	0.60
37	60	6	0.430	0.925	4.62	4.32	0.30	16.32	2446	5367	0.46
20	60	2	0.484	0.944	5.37	5.12	0.26	17.99	2782	6068	0.46
20	60	4	0.441	0.957	5.84	5.63	0.22	28.07	3158	4847	0.65
20	60	6	0.392	0.951	4.69	4.49	0.20	30.07	2645	4298	0.62
51	50	2	0.386	0.900	4.22	3.85	0.36	14.25	2270	6649	0.34

51	50	4	0.355	0.902	3.78	3.46	0.32	16.65	2124	5680	0.37
51	50	6	0.326	0.931	2.66	2.50	0.16	27.80	1600	2613	0.61
51	50	2	0.389	0.924	3.42	3.19	0.22	19.22	1887	3873	0.49
51	50	4	0.365	0.954	2.43	2.33	0.10	36.20	1417	1560	0.91
51	50	6	0.343	0.949	2.34	2.24	0.10	35.29	1398	1679	0.83
20	50	2	0.382	0.964	4.75	4.61	0.15	43.01	2744	2402	1.14
20	50	5	0.345	0.955	2.42	2.33	0.09	40.11	1450	1509	0.96
24	50	2	0.393	0.963	2.14	2.08	0.07	40.54	1220	1107	1.10
24	50	4	0.375	0.957	2.18	2.10	0.08	37.44	1258	1301	0.97
24	50	6	0.358	0.963	1.81	1.75	0.06	46.99	1074	914	1.18
24	40	2	0.294	0.963	3.26	3.16	0.10	62.22	2139	1587	1.35
24	40	4	0.267	0.958	2.63	2.53	0.09	62.39	1813	1436	1.26
63	40	3	0.293	0.903	2.57	2.35	0.21	22.47	1585	3603	0.44
63	40	6	0.262	0.913	2.08	1.92	0.16	29.50	1381	2508	0.55
37	30	2	0.220	0.982	0.72	0.71	0.01	191.66	571	158	3.63
37	30	7	0.205	0.975	0.90	0.88	0.02	148.50	746	277	2.70
32	20	5	0.138	0.962	0.48	0.46	0.02	158.62	521	209	2.49
32	20	9	0.127	0.490	0.92	0.49	0.43	6.60	557	14424	0.04
63	20	8	0.133	0.961	0.54	0.52	0.02	159.64	609	245	2.48

72	20	5	0.130	0.585	1.33	0.83	0.50	9.39	932	12888	0.07
63	10	8	0.067	0.938	0.11	0.10	0.01	211.61	227	75	3.02
79	10	8	0.069	0.419	0.205	0.094	0.111	10.08	185	4020	0.05

Table S7. Pervaporation results for PIM-Py membranes crosslinked with $Pd(OAc)_2$.

Membrane thickness (μm)	Feed initial toluene (vol%)	Time (h)	Feed toluene mole fraction	Permeate toluene mole fraction	Total flux (kg m^{-2} h^{-1})	Toluene flux (kg m^{-2} h^{-1})	DMSO flux (kg m^{-2} h^{-1})	Toluene/ DMSO separation factor	Toluene permeance (GPU)	DMSO permeance (GPU)	Toluene/ DMSO selectivity
31	77	2	0.676	0.959	7.12	6.87	0.25	11.15	3370	5092	0.66
20.5	77	2	0.671	0.959	9.51	9.18	0.33	11.44	4517	6760	0.67
38.3	77	2	0.681	0.945	5.41	5.16	0.25	8.11	2526	5304	0.48
